# Integrated Hydrophilic Interdigitated Network for Silicone Rubber via a Gradient Polarity Modification Strategy

**DOI:** 10.1002/advs.75378

**Published:** 2026-04-20

**Authors:** Lihui Zhang, Rui Wang, Pingru Su, Yao Kou, Zhengfeng Ma, Hui Tang, Mingying Wang, Lixin Wang, Chunyu Zhou, Shuanhong Ma, Yu Tang, Feng Zhou

**Affiliations:** ^1^ State Key Laboratory of Natural Product Chemistry Key Laboratory of Nonferrous Metal Chemistry and Resources Utilization of Gansu Province College of Chemistry and Chemical Engineering Lanzhou University Lanzhou P.R. China; ^2^ State Key Laboratory of Solid Lubrication Lanzhou Institute of Chemical Physics Chinese Academy of Sciences Lanzhou P.R. China; ^3^ Shandong Laboratory of Advanced Materials and Green Manufacturing At Yantai Yantai Zhongke Research Institute of Advanced Materials and Green Chemical Engineering Yantai P.R. China

**Keywords:** gradient polarity modification, hydrophilic functionalization, interdigitated network, multifunctional materials, silicone rubber

## Abstract

Enabled by excellent biocompatibility and functional designability, hydrophilic flexible functional materials are gaining traction in fields like flexible electronics and implantable/interventional medical devices. Silicone rubber (SR), as a conventional high‐performance material, exhibits outstanding flexibility, fatigue resistance, and biocompatibility, making it an ideal substrate for constructing such functional materials. However, its inherent hydrophobicity and low surface energy severely limit compatibility with polar modifiers and hinder further functionalization. To address this, we developed a universal in situ modification strategy based on a “gradient polarity modification” concept. By establishing a polarity transition ladder between SR and strongly polar hydrophilic materials (e.g., quaternary ammonium and zwitterionic compounds), this method successfully achieves a hydrophilic interdigitated SR network and enables modification from the surface to the bulk. The resulting materials exhibit a combination of superior properties, including persistent bulk hydrophilicity, remarkable aqueous lubrication, and maintained mechanical robustness. By demonstrating the successful fabrication of a lubricative/antibacterial catheter and a long‐lasting lubrication meniscus, this strategy proves to be highly designable in function and directly applicable for modifying pre‐formed SR devices. Its exceptional responsiveness, as confirmed by motion capture, underscores a significant potential for use in advanced sensing applications.

## Graphical abstract

This work overcomes the long‐standing challenge of hydrophilic modification of silicone rubber via a gradient polarity modification strategy. By constructing a hydrophilic interpenetrating network within cured SR, simultaneous surface‐to‐bulk modification is achieved while enabling monitoring through a Eu^3+^ complex fluorescent tracer. The resulting multifunctional material integrates the advantages of both components, demonstrating promising potential for biomedical and flexible electronics applications.

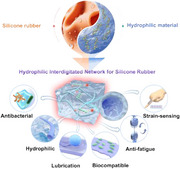

## 1 Introduction

The construction of Integrated Hydrophilic Interdigitated Networks, which achieves exceptional versatility through performance complementarity, customizable functionality, and structural integration of diverse components, has been demonstrated as an effective strategy for fabricating multifunctional materials [1, 2, 3, 4, 5, 6, 7]. With outstanding properties including flexibility, fatigue resistance, thermal stability, chemical inertness, and biocompatibility [8, 9, 10], the silicone rubber (SR) network is an ideal material for diverse applications, including biomedical materials, flexible electronics [11, 12, 13, 14, 15], microfluidic devices [16, 17, 18], and dynamic seals. However, the intrinsic hydrophobicity of the siloxane backbone poses a major constraint, resulting in high interfacial friction, elevated electrical resistance, and a pronounced susceptibility to biofouling [19, 20]. These limitations impede the effectiveness of SR in applications requiring hydrophilic, lubricious interfaces, such as biomimetic interfaces [21, 22], sensors [23, 24, 25, 26], and water‐lubrication system [27, 28]. Therefore, hydrophilic modification is indispensable for unlocking the full potential of silicone rubber, ideally while retaining its intrinsic advantages and incorporating the multifunctionality characteristic of hydrophilic materials.

Achieving durable hydrophilic modification within cured SR remains challenging due to its inherent low surface energy, weak polarity, and high chemical stability, which collectively hinder adhesion, miscibility, and grafting with polar modifiers [29]. Current modification strategies for SR face significant limitations: surface modification techniques, such as plasma treatment and coatings [30], often suffer from poor durability and are difficult to apply to bulk modification. Physical blending enables bulk modification but faces the persistent challenge of phase separation [31, 32, 33, 34, 35]. While chemical grafting represents an effective route to stable functional networks, it typically relies on custom‐designed precursors and is hindered by polarity mismatch [36]. While radiation‐induced grafting has proven to be an effective pathway for modifying commercial SR [37, 38], its widespread adoption is hindered by the limited availability of radiation sources and is further complicated by unresolved compatibility issues with strongly polar functional structures. Consequently, a pressing need remains for a generalized strategy to overcome the fundamental polarity mismatch between hydrophilic modifiers and the SR network and achieve robust, deep bulk functionalization under mild conditions.

**FIGURE 1:**
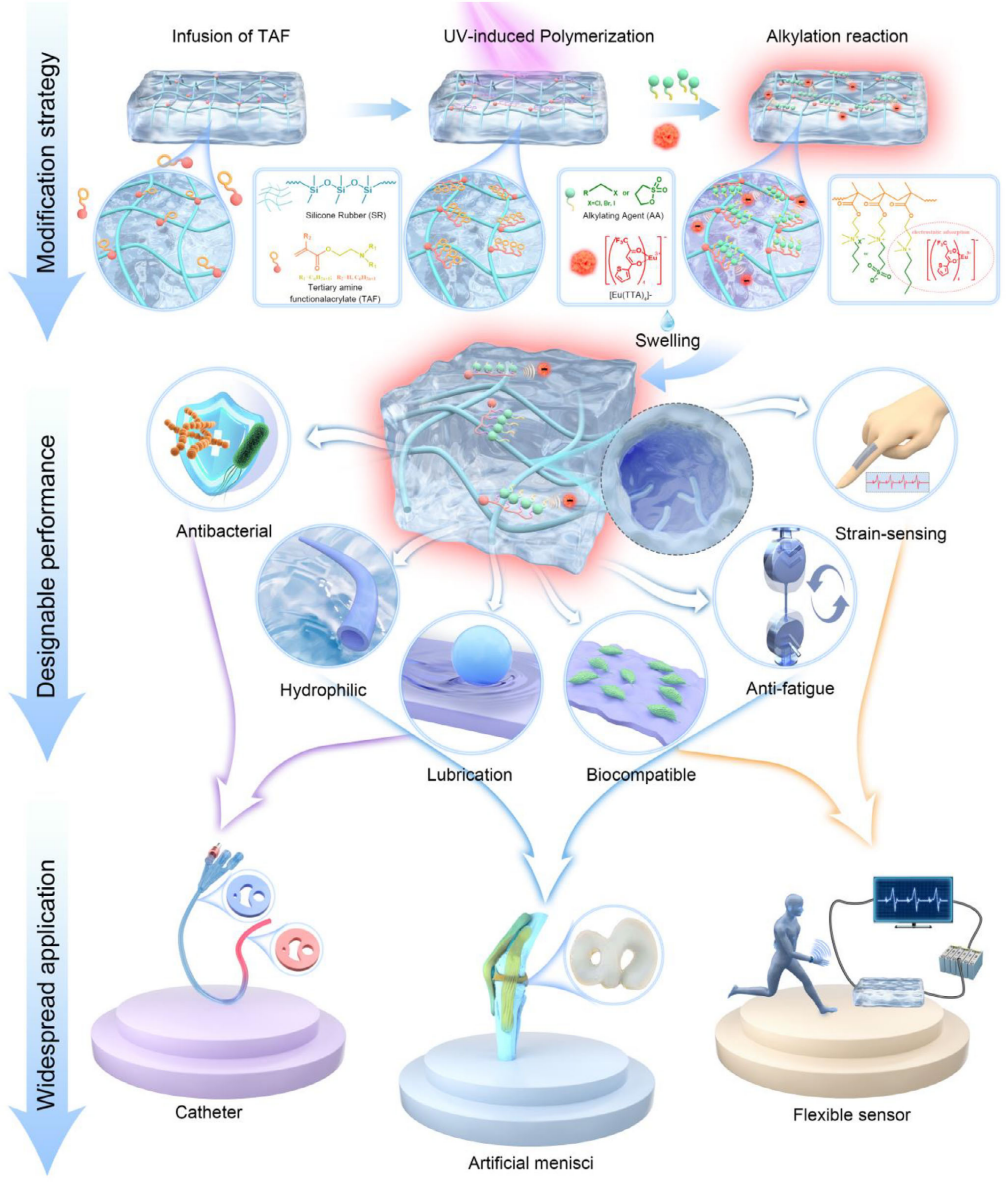
The “gradient polarity modification” strategy for constructing of hydrophilic interdigitated SR networks and their applications.

Herein, we propose a “gradient polarity modification” strategy to fabricate a hydrophilic interdigitated SR network directly from commercial pre‐cured SR. The core design concept employs a stepwise increase in polarity to mitigate the thermodynamic incompatibility between the hydrophobic SR and polar modifiers. This strategy inherits the conceptual framework of conventional surface stepwise grafting modification [39, 40] while incorporating precise tailoring of functional molecule polarity, extending the scope of interfacial regulation from surface modification to bulk‐phase design. In contrast to approaches that employ a polarity gradient transition layer to alleviate interfacial stress between dissimilar phases [41], our method covalently incorporates polar molecules into the SR network through a sequentially constructed polarity gradient, resulting in a macroscopically homogeneous structure. Collectively, this approach offers an additional route for SR functionalization, achieving simultaneous hydrophilic modification of the surface and bulk via gradient polarity engineering. The implementation of this strategy consists of a three‐step process (Figure 1): 1) infusion of a moderately polar and highly reactive tertiary amine‐functional acrylate (TAF) into the SR network; 2) UV‐induced in situ polymerization and grafting enable the construction of a polarity transition ladder; and 3) solvent‐mediated incorporation of a strongly polar alkylating agent to induce alkylation, forming deeply embedded ionic domains. To enable direct visualization of the alkylation layer, a hydrophobic Eu^3+^ complex was incorporated as a fluorescent tracer [42, 43, 44]. The alkylation degree and spatial extent of modification were precisely controlled by varying the reaction time, forming a hydrophilic interdigitated network that ensured long‐term stability and bulk hydrophilicity.

To explore the versatility of our strategy beyond the initial demonstration, SR was modified with selected alkylating agents (AAs), illustrating the functional tunability afforded by this method and highlighting its potential for broader applicability. The resulting SR network structures exhibit exceptional hydrophilicity, with a water absorption rate exceeding 100%, and can be engineered to possess several advanced functional characteristics: excellent lubrication performance, evidenced by an ultralow bulk coefficient of friction (COF) of 0.035 and a stable surface COF of approximately 0.1 even after 10 000 friction cycles; 100% antibacterial efficacy against *E. coli* and *S. aureus*; and the successful incorporation of highly polar, zwitterionic hydrophilic structures with excellent biocompatibility, laying the foundation for applications in biomedical materials. Importantly, our bulk‐modification strategy imparts ionic conductivity to insulating SR, thereby conferring sensitive and stable strain‐sensing capabilities. Leveraging the flexibility and multifunctionality of this strategy, we successfully functionalized complex, pre‐formed SR devices such as lubricative/antibacterial urinary catheters, long‐lasting lubrication/biocompatibility artificial menisci, and flexible/sensitive motion sensors. Predictably, this integrated hydrophilic interdigitated SR network would find broad applications in areas such as advanced flexible sensors and implantable biomedical devices.

## 2 Results and Discussion

### 2.1 Design and Characterization of the Hydrophilic Interdigitated SR Network

**FIGURE 2:**
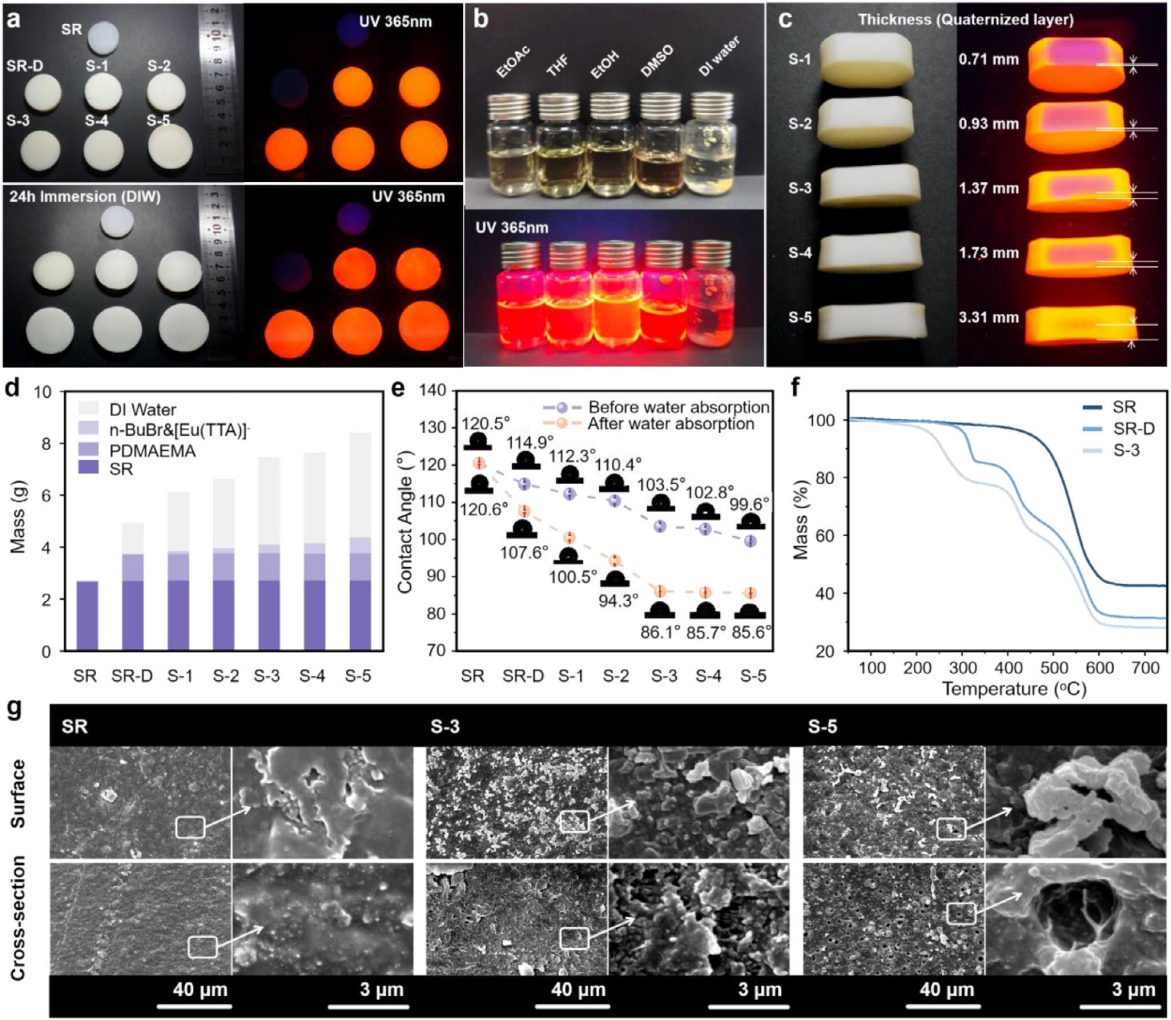
Composition, structure and properties of hydrophilic interdigitated SR networks. (a) Photos of hydrophilic interdigitated SR networks in visible and ultraviolet light. (b) Photos of the solubility of [Eu(TTA)_4_]^–^ in different solvents and their luminescence under UV light. (c) Photos of the cross‐section of hydrophilic interdigitated SR networks in visible and ultraviolet light. (d) Component content of SR networks before and after modification. (e) Contact Angle of SRs before and after modification. (f) *Tg* curves of SRs before and after modification. (g) SEM images of SRs before and after modification.

Successful implementation of the gradient polarity modification strategy yielded a series of hydrophilic interdigitated SR networks with visually tunable degrees of modification (Figure 2a). The commercial SR was functionalized with 2‐(dimethylamino) ethyl methacrylate (DMAEMA) as the TAF (Figure S1), and n‐BuBr as the alkylating agent to construct hydrophilic networks modified with quaternary ammonium polar structures.

By co‐penetrating with the alkylating agent and electrostatically adsorbing onto the resultant quaternary ammonium groups, the Eu^3+^ complex [Eu(TTA)^4^]^–^ (Figures S2 and S3) served as a fluorescent indicator to mark the quaternization reaction front. Its strong luminescence (Figures S4 and S5), solubility in organic solvents, and water immiscibility helped visualize the effective alkylation layer thickness (Figure 2b). As illustrated in Figure 2c, under 365 nm UV irradiation, regions undergoing effective alkylation exhibited intense red fluorescence that persisted after water absorption. Cross‐sectional imaging quantified the alkylation layer, which grew with reaction time from 0.71 mm (S‐1, 30 min) to 3.37 mm (S‐5, 5 h), confirming its use in tracking reagent penetration and hydrated layer thickness.

The composition of the modified networks was qualitatively analyzed by FTIR (Figure S6) and quantitatively assessed by gravimetric analysis (Figure 2d). Gravimetry indicated a DMAEMA grafting ratio of 38.5 ± 0.5 wt.%. Subsequent quaternization further increased the mass increment from 4.3 wt.% (S‐1, 30 min) to 22.9 wt.% (S‐5, 8 h). This significantly improved the hydrophilicity of the networks, as reflected by increased water absorption rates and capacities (Figure S7). With increasing quaternization degree, the water uptake climbed from 0 wt.% (pristine SR) to 81.4 wt.% (S‐1), rapidly exceeded 100% (122.7 wt.%, S‐3), and finally at 146.2 wt.% (S‐5).

Surface hydrophilicity, evaluated by water contact angle (WCA) measurements (Figure 2e), decreased progressively from >120° for pristine SR to 114.9° after DMAEMA grafting. With increasing quaternization (samples S‐1 to S‐5), the WCA further declined to 99.6°. After 7 days of water immersion, the WCA of pristine SR remained above 120°, whereas all modified samples exhibited lower WCAs. The post‐immersion WCAs initially decreased to 86.1° (S‐3) and then stabilized at 85.7° (S‐4) and 85.6° (S‐5). The enhanced hydrophilicity after immersion is attributed to the migration and enrichment of strongly polar groups at the surface, where they immobilize water molecules to form a hydrated layer, thereby markedly improving droplet spreading and wetting.

Thermogravimetric analysis (TGA, Figure 2f) results indicated that the pristine SR exhibited single‐stage decomposition starting at 450°C, yielding a residual mass of 42.5 wt.% at 750°C. In contrast, SR‐D displayed three decomposition stages: an initial mass loss of 15 wt.% beginning around 275°C, corresponding to the degradation of tertiary amine groups via β‐hydrogen elimination or ester cleavage; a second mass loss of 20 wt.% starting at 330°C, attributed to PDMAEMA backbone decomposition; and a final stage from 460°C, representing decomposition of the silicone matrix. The quaternized sample (S‐3) also exhibited three‐stage decomposition, but with shifted onset temperatures: Stage 1 commenced significantly earlier at 217°C due to Hofmann elimination of quaternary ammonium groups (‐N^+^(CH_3_)_2_(C_4_H_9_)Br^−^), confirming reduced thermal stability after quaternization; Stage 2 started at 315°C, assigned to cleavage of unquaternized side chains and backbone segments [45, 46]; and Stage 3 began at 450°C, reflecting decomposition of the silicone matrix. Compared to the DMAEMA‐modified sample prior to quaternization, S‐3 showed increased mass loss in the corresponding stages, primarily due to thermal decomposition of the additional alkyl chains introduced during quaternization.

Scanning electron microscopy (SEM) of surfaces and cross‐sections (lyophilized after 7‐day immersion to preserve the hydrated morphology) revealed critical microstructural changes elucidating the evolution of the material structure (Figure 2g; Figure S8). The modification process markedly increased surface roughness due to PDMAEMA grafting. Cross‐sectional images clearly evidenced this increased roughness and further showed that quaternization induced the formation of 2–5 µm micropores containing dendritic polymer aggregates. These morphological transformations are attributed to the substantially enhanced hydrophilicity and water absorption capacity, wherein water molecules preferentially accumulate in regions concentrated with quaternary ammonium groups, facilitating microchannel formation. These channels constitute the structural basis for high water absorption, while directly demonstrating significant reorganization of the SR network.

### 2.2 Mechanical and Strain‐Dependent Electrical Properties

**FIGURE 3:**
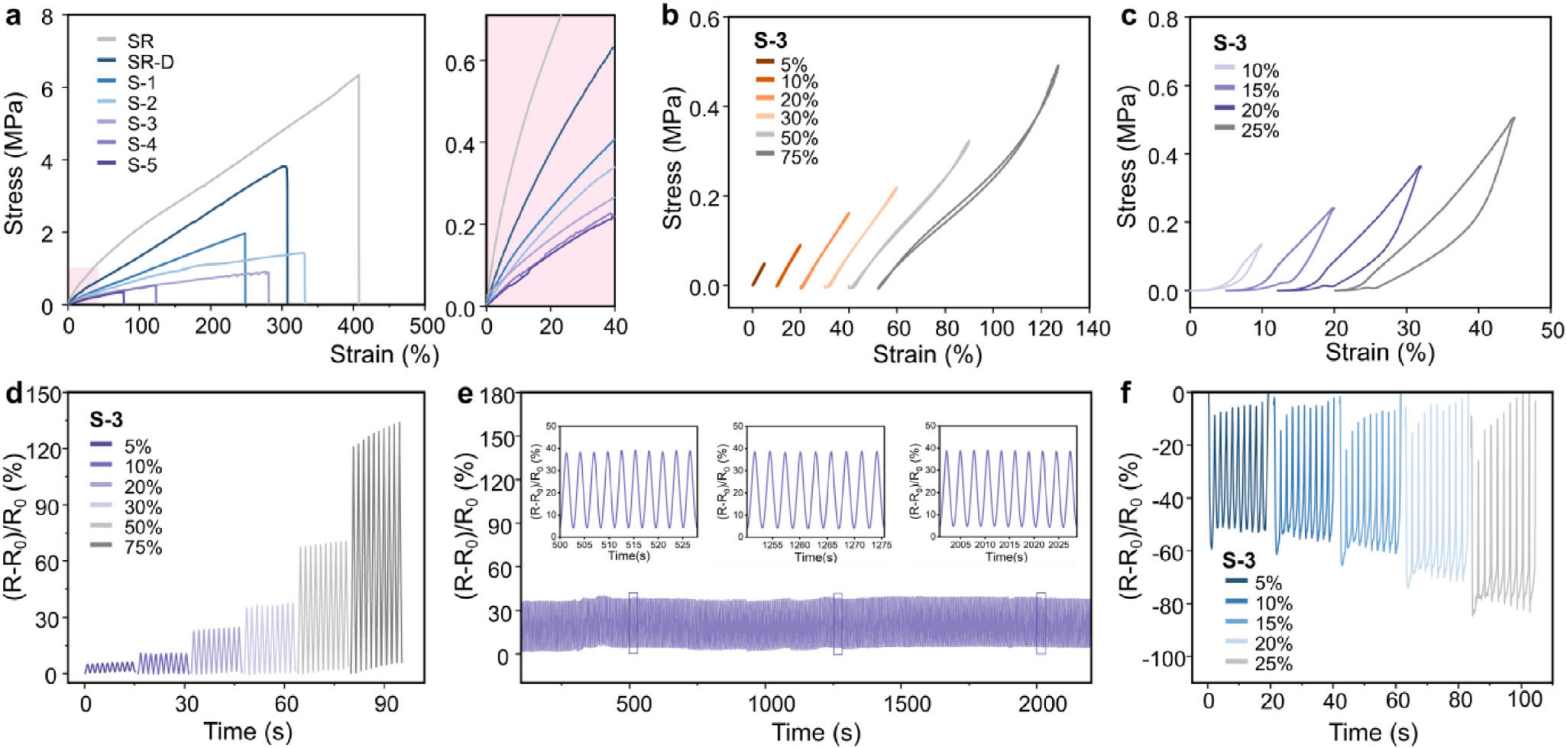
Mechanical and strain‐dependent electrical properties of pristine SR and hydrophilic interdigitated SR networks. (a) Tensile mechanical properties. (b) Cyclic test of tensile properties (S‐3). (c) Cyclic compression performance tests (S‐3). (d) Cyclic tensile sensing test with different stretches (S‐3). (e) Cyclic stability of tensile sensing (1000 cycles). (f) Cyclic compression sensing test with different compressibility (S‐3).

In order to quantify the mechanical properties of the hydrophilic interdigitated SR networks, various mechanical tests were performed. Hydrophilic modification significantly impacts mechanical properties by altering the pristine network structure and introducing substantial water absorption. Tensile testing (Figure 3a) revealed that, compared to pristine SR, both the elastic modulus and fracture strength of quaternized samples decreased monotonically with extended quaternization time, confirming an inverse correlation between enhanced hydrophilicity and mechanical integrity. Despite an overall declining trend in elongation at break, the value notably increased to 330% for the 1‐h quaternized sample (S‐2), exceeding that of the non‐quaternized SR‐D, before decreasing again to 123% (S‐4) and 85% (S‐5). This mechanical deterioration stems from three interconnected mechanisms: a network dilution effect where water infiltration increases chain separation and effective segment length between crosslinks, reducing crosslink density and load‐bearing capacity; swelling‐induced chain extension decreasing entanglement density and facilitating chain slippage, explaining the relatively higher elongation in S‐2 [47, 48]; and structural damage from excessive modification, evidenced by non‐uniform swelling, internal stress generation, and microcrack/void formation (consistent with prior SEM observations), creating stress concentration points that accelerate mechanical degradation.

Cyclic tensile testing (Figure 3b) demonstrated strain‐dependent hyperelasticity: below 50% strain, loading‐unloading curves overlapped completely, indicating elasticity‐dominated deformation; beyond 50% strain, irreversible mechanisms activated, with hysteresis loops emerging at 75% strain due to network damage and chain slippage. Compression cycling (Figure 3c; Figure S9) test results indicated that the pristine silicone exhibited hysteresis at 10% strain. The modified samples (S‐3) showed a reduced modulus, and their enlarged hysteresis loops suggested enhanced internal friction during deformations without substantial network damage. Collectively, these findings demonstrate that quaternization duration enables synergistic optimization of hydrophilicity and mechanical performance. Moderately modified samples (1‐2 h) maintain high elongation (>280%) with stable hysteresis, suitable for sealing applications requiring cyclic deformation tolerance. Highly hydrophilic samples (4‐5 h) exhibit rapid energy dissipation characteristics, showing promise for underwater damping and shock absorption applications.

The bulk‐surface hydrophilic modification introduces abundant ionic structures into the SR material through ionic polymer grafting and confined filling, coupled with significant water‐absorption capacity. These combined effects give rise to ionic conductivity, and the resulting resistive response forms the basis for its application as a flexible strain sensor [49, 50]. To quantify the strain‐dependent electrical properties, we designed and built a testing apparatus for both tensile and compressive modes (Figure S10). Results demonstrate that a strain sensor utilizing this modified material as its core exhibits a significant strain dependence within the 5%‐75% tensile strain range: the relative resistance change ((R‐R_0_)/R_0_) increased from 5% to 125% (Figure 3d; Video S1). Leveraging the superior fatigue resistance of the material (Figure S11), a 1 000‐cycle test was further conducted under a fixed tensile strain of 50% (Figure 3e). The sensor demonstrated excellent cyclic stability and durability, with the signal amplitude of (R‐R_0_)/R_0_ showing no significant changes during the initial, middle, or later stages of cycling. Furthermore, the sensing behavior of the sensor in compression mode was investigated (Figure 3f; Video S2). Within the 5%‐25% compressive strain range, the relative resistance change (R‐R_0_)/R_0_ ranged from approximately ‐50% to ‐80%. This negative change indicates a decrease in the material's resistance under compressive deformation, validating the potential of this modified SR as a core sensing material in compression mode and demonstrating its ability to effectively sense and distinguish between tensile and compressive deformation modes.

### 2.3 Tribological Properties

**FIGURE 4:**
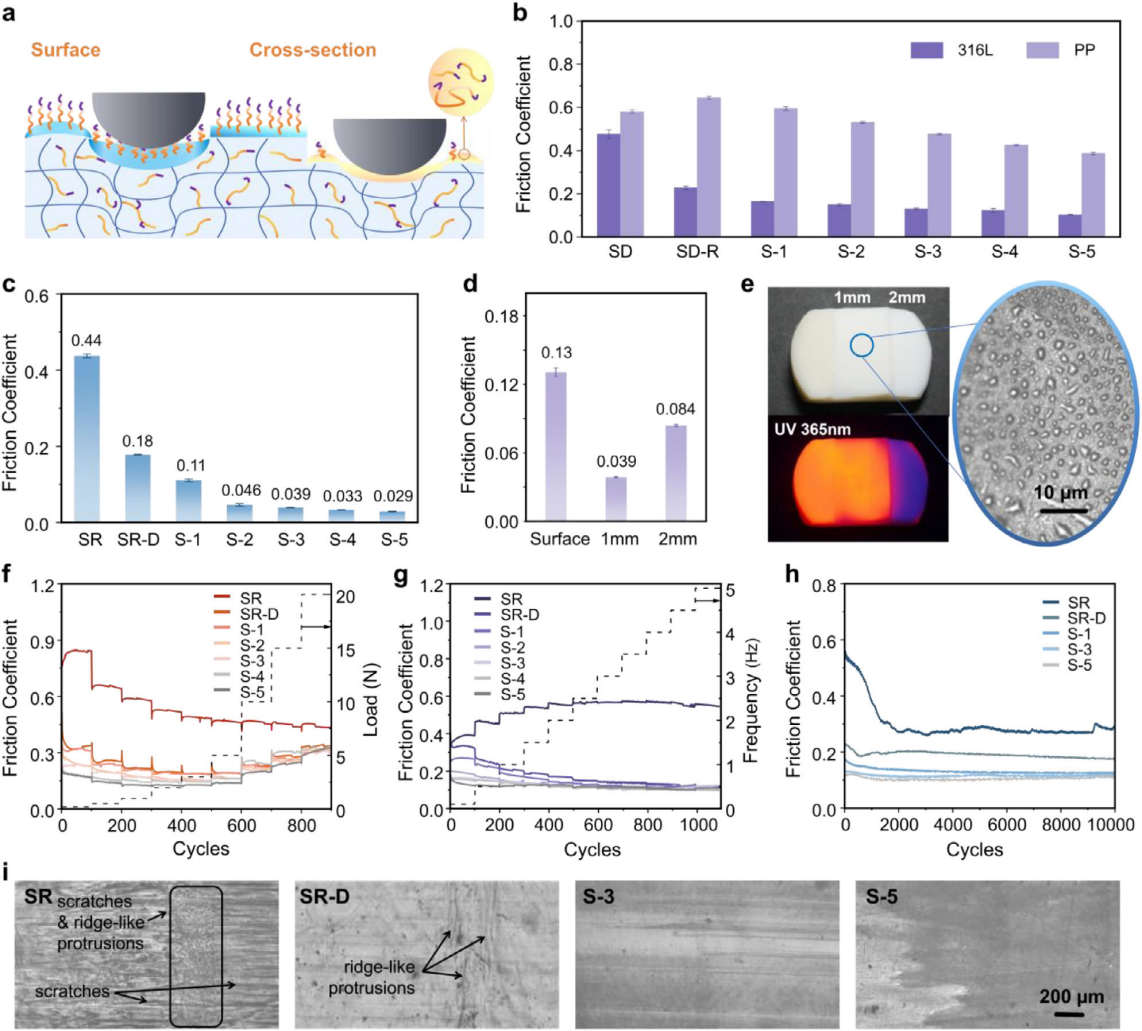
Tribological properties of pristine SR and hydrophilic interdigitated SR network. (a) Schematic diagram of the lubrication performance of the hydrophilic interdigitated SR network. (b) COFs of hydrophilic interdigitated SR network with different materials. (c) Bulk COFs of hydrophilic interdigitated SR networks. (d) The COFs of hydrophilic interdigitated SR network surfaces and sections at different depths. (e) Photos of different depth sections in visible and UV light (365 nm), optical microscope photograph of the section (1 mm). (f) COFs under different loads. (g) COFs under different frequencies. (h) COFs with 10 000 cycles. (i) Photos of the abrasion marks after 1,0000 cycles.

Water lubrication ability represents a crucial performance characteristic of hydrophilic polymer materials [51, 52, 53]. In this study, the water‐lubricated tribological behavior of pristine SR and hydrophilic interdigitated SR networks was systematically evaluated using a CSM tribometer. Based on the structural features of the modified materials, we propose two lubrication mechanisms (Figure 4a): i) surface hydration lubrication, originating from the formation of a stable hydration layer on the hydrophilic surface; and ii) bulk lubrication, where, in addition to covalently grafted chains, hydrophilic homopolymer fillers function predominantly as free lubricants. The tribological performance of the materials was tested on the surface, in the bulk, and under varying operating conditions.

Surface hydration lubrication: The coefficient of friction (COF) under water lubrication was measured against different counterfaces (Figure S12). For hydrophilic 316L steel, pristine SR exhibited a relatively high COF (µ = 0.48); after DMAEMA grafting (SR‐D), the COF decreased to µ = 0.23; further quaternization (S‐5) achieved µ = 0.089 (Figure 4b), primarily because the enhanced surface hydrophilicity promotes hydration layer formation while effectively reducing interfacial adhesive friction (Figure S13). For hydrophobic PP counterfaces, pristine SR showed a high COF (µ = 0.58) due to hydrophobicity and adhesion. After DMAEMA grafting, the COF unexpectedly increased to 0.64, likely due to hydration‐induced swelling or softening caused by surface hydrophilization, which increased the real contact area. Further quaternization reduced the COF to µ = 0.49. In summary, hydrophilic modification improves the surface hydrophilicity and forms a hydration lubrication layer, but the effectiveness of this strategy depends on the nature of the counterface—the hydration layer can stably exist and exert its excellent lubrication only on hydrophilic interfaces.

Bulk lubrication: In addition to surface effects, the modified materials exhibit a unique bulk lubrication characteristic, which originates from the dual lubricating action of hydrophilic grafted polymer chains and homopolymer fillers. To characterize the bulk lubrication properties, COF measurements were performed on horizontal cross‐sections at a depth of 1 mm from the surface (Figure 4c). Notably, the COF on the cross‐section was significantly lower than that on the surface: SR‐D achieved µ = 0.18 at 1 mm depth; with increasing degree of quaternization, the COF further decreased, reaching µ = 0.029 for S‐5 – a 93% reduction compared to pristine SR. Depth‐dependent COF testing of sample S‐3 (Figure 4d) gave values of µ = 0.13 (surface), µ = 0.039 (1 mm depth), and µ = 0.084 (2 mm depth). UV light imaging (Figure 4e) confirmed that after 2 h of treatment, the AA reagent penetrated to a depth of 1 mm in S‐3 but did not reach 2 mm, thereby establishing a clear structure‐property relationship. Optical microscopy of the cross‐section revealed exuded droplets, and FTIR identified the exudate composition (Figure S14) as PDMAEMA, confirming that the bulk lubrication paradigm of the modified SR is a synergistic effect of grafted hydrophilic polymers and controlled exudation of lubricating fluid. This provides a new approach for designing advanced functional elastomers with exceptional and durable tribological properties.

Tribological performance under varying operating conditions: Frictional performance exhibits a strong dependence on load, sliding frequency, and cycle number; therefore, systematic evaluation of the response characteristics under different operating conditions is crucial. As shown in Figure 4f–i, the tribological characteristics of the modified silicone rubber were further revealed through tests under varying load (0.1‐20 N), frequency (0.1‐5 Hz), and 10 000 cycles.

Load dependence (Figure 4f): Pristine SR exhibited a high COF of 0.85 at 0.1 N, which gradually decreased with increasing load and stabilized at 0.48 above 5 N, consistent with the classical tribological behavior of rubber materials [54]. After hydrophilic modification, the COF at 0.1 N decreased with increasing degree of quaternization from 0.30 to 0.18, reached a minimum in the intermediate load region (2‐3 N), and was around 0.30 at a high load of 20 N. Thus, the modified materials exhibited an overall trend of first decreasing and then increasing COF with load, outperforming pristine SR across the entire load spectrum (0.1‐20 N), with particular advantages in the low‐load regime (≤ 3 N). This phenomenon can be attributed to the stable hydration layer formed on the hydrophilic surface, which effectively reduces adhesive friction at low loads; however, excessively high loads cause thinning or even rupture of the hydration film, leading to an increase in COF [55, 56].

Frequency dependence (Figure 4g): For pristine SR, the COF was below 0.40 at 0.1 Hz, increased to a peak of about 0.60 at 2.5 Hz, and then stabilized at higher frequencies. This behavior originates from the competition between adhesion‐dominated friction at low frequencies and enhanced viscoelastic dissipation at high frequencies [57]. The modified systems displayed an opposite trend: SR‐D decreased from 0.35 at 0.1 Hz to 0.10 at 5 Hz, representing a 71% reduction in peak friction relative to pristine SR. Quaternized samples exhibited COF values between 0.11 and 0.25 at 0.1 Hz, and stable values of 0.10‐0.11 at 5 Hz, with an overall friction reduction of about 80% across the whole frequency range. Moreover, with increasing hydrophilicity (S‐3 to S‐5), the sensitivity of COF to frequency significantly decreased (Δµ < 0.05). This transition is primarily attributed to the formation of a hydration layer that provides effective water lubrication at low frequencies, and to the covalently grafted network structure that endows the material with excellent resistance to shear‐induced dehydration, ensuring the stability of the hydration layer under high‐shear conditions.

Durability (Figure 4h–i): Pristine SR had an initial COF of 0.58, which stabilized at 0.30 after a running‐in period of about 2,000 cycles. The wear scar exhibited typical rubber wear features, i.e., grooves parallel to the sliding direction and ridges perpendicular to it [58]. SR‐D stabilized at a COF of 0.20 after about 600 cycles, with shallower grooves. Quaternized samples (S‐3 to S‐5) reached a stable low friction level (µ ≈ 0.10) after only about 500 cycles and maintained this performance for 10 000 cycles without significant decay; the wear scar surfaces showed no ridges or obvious grooves, indicating a fundamental change in the wear mechanism. This exceptional performance is attributed to the optimized surface hydration that maintains a stable water lubrication layer, together with significantly reduced bulk viscoelastic dissipation [59].

In summary, hydrophilic modification (especially quaternization) significantly enhances the lubricity and frictional stability of the materials under wide load ranges, wide frequency ranges, and long‐term cycling conditions.

### 2.4 Exploring the Applicability of the Method

**FIGURE 5:**
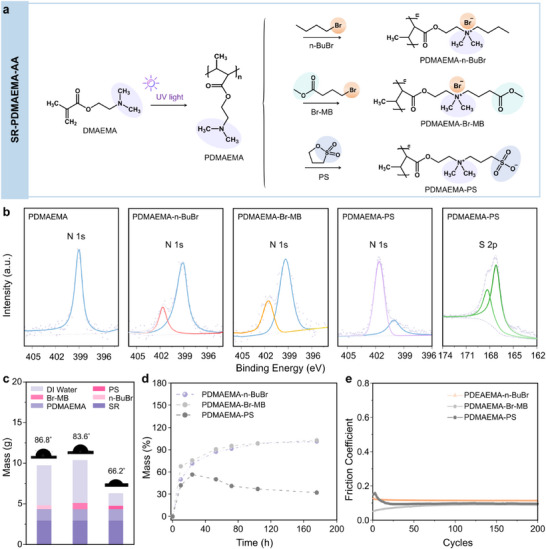
Exploring the applicability of the method. (a) Preparation of PDMAEMA‐AA modified SR. (b) XPS survey spectrum of SR‐PDMAEMA‐AA. (c) Composition and surface water contact angle of modified SR. (d) Water absorption performance of modified SR. (e) COF of modified SR under water lubrication conditions.

In this study, tertiary amine‐functionalized (TAF) monomers were employed as reactive modifiers (Figures S15 and S16), enabling the construction of distinct ionized networks via nucleophilic substitution reactions to impart tailored functionality. To explore the versatility of this gradient polarity modification strategy (Figure 5a) for simultaneous bulk and surface hydrophilic ionization of silicone rubber (SR), we additionally selected two alkylating agents: methyl 4‐bromobutyrate (Br‐MB) and 1,3‐propanesultone (PS). Specifically, Br‐MB reacts with the tertiary amine groups via an SN2 mechanism to form quaternary ammonium salts, introducing permanent positive charges that confer antimicrobial activity and tunable wettability. Meanwhile, PS undergoes ring‐opening SN2 reaction to generate sulfobetaine‐type zwitterionic structures (inner salts), achieving overall charge neutrality with locally balanced charges, which endows the material with excellent antifouling properties, biocompatibility, and ultrahigh hydrophilicity.

The successful construction of quaternary ammonium and zwitterionic structures was characterized by X‐ray photoelectron spectroscopy (XPS, Figure 5b). For the tertiary amine‐modified sample (SR‐PDMAEMA), the N 1s spectrum exhibits a peak at approximately 399.1 eV, characteristic of tertiary amine nitrogen. Upon alkylation with n‐BuBr and Br‐MB, an additional N 1s peak is observed at 401.6 eV, which is characteristic of quaternary ammonium species [60]. After reaction with 1,3‐propanesultone (PS) to form the sulfonic zwitterion, the N 1s peak appears at approximately 401.6 eV, characteristic of the quaternized nitrogen in zwitterionic structures. Concurrently, the S 2p signal is observed at approximately 168 eV, corresponding to the sulfonate group (SO_3_^–^) [61], confirming the successful formation of the zwitterionic moiety. As evidenced by the ratio of tertiary to ionized nitrogen species derived from the N1s spectra, under identical reaction conditions (60°C, 2 h), PS led to a higher degree of tertiary amine conversion compared with the other two alkylating agents. Collectively, these XPS results provide direct spectroscopic evidence for the successful implementation of each alkylation step and the precise chemical control achieved through the gradient polarity modification strategy.

The hydrophilicity of SR‐PDMAEMA after alkylation is presented in Figure 5c,d. The n‐BuBr‐modified sample exhibited a water absorption of 102.0% and a WCA of 86.8°. Owing to its inherent polarity, Br‐MB yielded superior hydrophilicity compared to the n‐BuBr‐modified counterpart, with a water absorption of 102.5% and a water contact angle (WCA) of 83.6°, alongside faster absorption kinetics, achieving 67.5% water uptake within 10 h. While PS generated a sulfobetaine zwitterionic structure that imparted excellent surface hydrophilicity (WCA: 66.2°), the resulting excessive hydrophilicity induced notable network disruption upon prolonged water exposure, as evidenced by surface dissolution and mass loss after 24 h. DMAEMA‐grafted networks after reaction with PS form a sulfobetaine structure (‐N^+^(CH_3_)_2_‐(CH_2_)_3_‐SO_3_^–^). As a strongly hydrating zwitterion, this structure undergoes significant swelling in pure water, generating substantial stretching forces and interfacial shear stress. At high grafting densities, the cumulative stress exceeds the strength of the silicone rubber network, leading to network disruption at the grafting points and surface peeling [62]. Water lubrication performance directly correlated with the achieved hydrophilicity (Figure 5e). Notably, SR‐PDMAEMA alkylated with Br‐MB and PS achieved excellent lubrication, with coefficients of friction (COF) of 0.08 and 0.10, respectively, outperforming the n‐BuBr‐modified reference (0.11).

These results confirm the potential of this strategy to realize distinct functional properties by selecting alkylating agents with tailored reactivity and polarity, thereby providing an approach to meet diverse application‐specific requirements.

### 2.5 Application Validation

**FIGURE 6:**
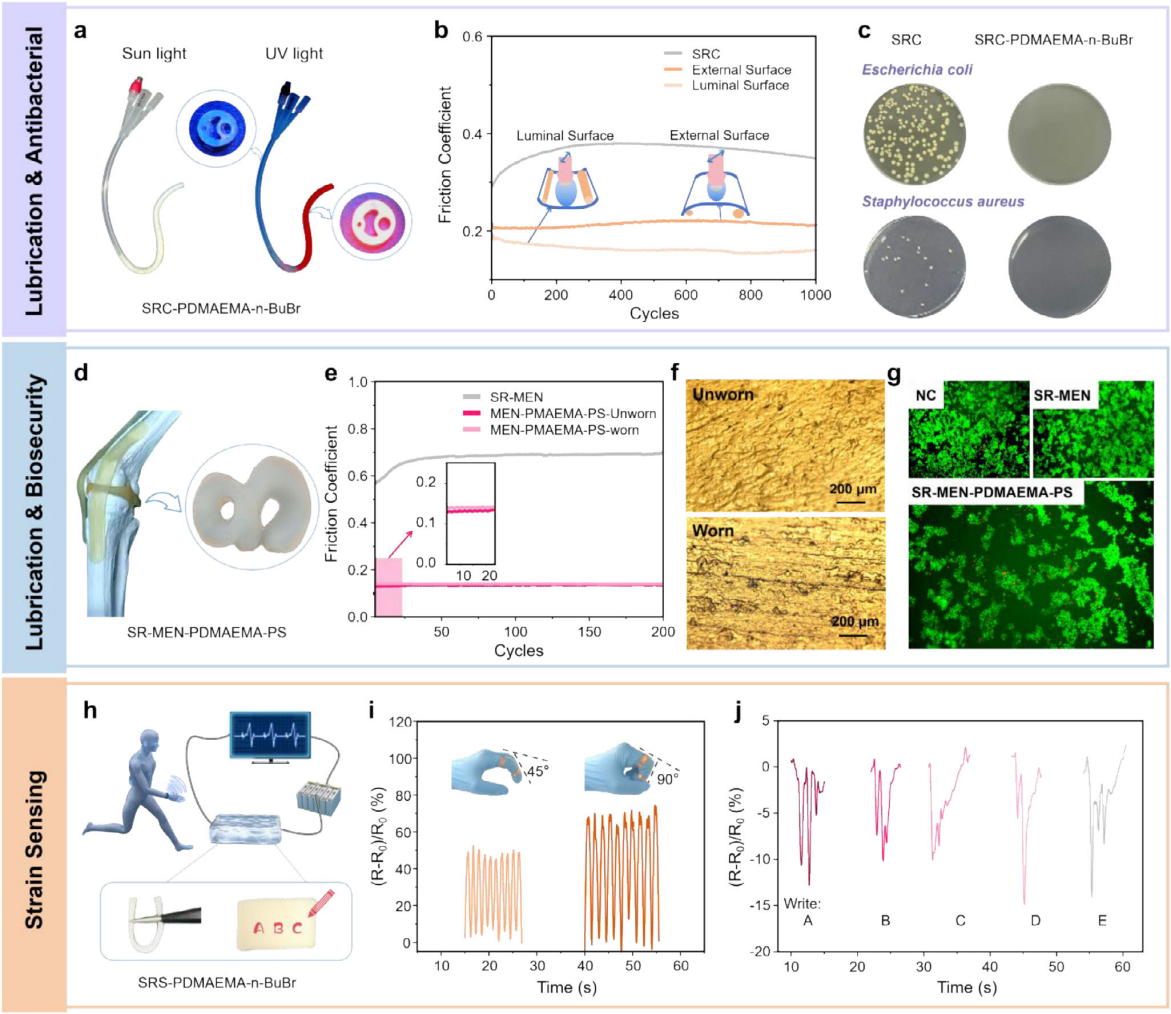
Application validation of SR with synchronous strong polar hydrophilic surface‐bulk modification. (a) Modified SRC (SRC‐PDMAEMA‐n‐BuBr). (b) Frictional performance of the modified SRC. (c) Antibacterial properties of the modified SRC. (d) Modified SR‐MEN (SR‐MEN‐PDMAEMA‐PS). (e) Hydrophilic lubricity of the modified SR‐MEN. (f) Surface optical micrograph of the modified SR‐MEN. (g) Cytocompatibility of the modified SR‐MEN. (h) Modified SRS (SR‐PDMAEMA‐n‐BuBr). (i) Sensing performance test for finger sensor. (j) Signal responses when writing “A, B, C, D, E, F”.

To further validate the application potential of the gradient polarity modification method, we applied it to complex structures such as a triple‐lumen silicone rubber catheter (SRC) and a silicone rubber meniscus (SR‐MEN). The catheter was subjected to modification using the “DMAEMA + n‐BuBr + [Eu(TTA)_4_]^–^” system. Cross‐sectional morphology (Figure 6a) revealed that both the luminal and external surface were modified while the millimeter‐scale microchannel structure remained intact. Friction tests (Figure 6b) showed that the COFs under aqueous lubrication decreased significantly from 0.36 to 0.21 (external surface) and 0.16 (luminal surface), indicating markedly improved hydrophilic lubricity. The luminal surface exhibited superior modification effectiveness due to reduced oxygen inhibition during photopolymerization. Due to the introduced quaternary ammonium structures (Figure S17), it demonstrated a 100% inhibition rate against both *E. coli* and *S. aureus* (Figure 6c) in an evaluation critical for catheter applications. The applicability to complex structures was evaluated using a silicone meniscus modified with the “DMAEMA + PS” system (Figure 6d). The surface COF in an aqueous environment dropped dramatically from 0.68 to 0.13 (Figure 6e). To assess lubricating durability, artificial wear was induced by sandpaper abrasion (Figure 6f). The worn surface maintained a low COF of 0.14, confirming that the synchronous surface‐to‐bulk modification imparts durable lubricating properties. Cytotoxicity tests showed no significant increase in cell mortality compared to the negative control (NC) and unmodified SR‐MEN, indicating favorable biocompatibility (Figure 6g).

Based on the strain‐sensing capability, a flexible SR sensor (SRS) was fabricated (modified with the “DMAEMA + n‐BuBr” system, Figure 6h). To evaluate its performance in wearable applications, the sensor was employed to monitor finger‐bending motions (Figure 6i). The electrical response showed a clear correlation with the bending angle: a 45° bend induced a relative resistance change ((R‐R_0_)/R_0_) of approximately 50%, which increased to about 70% at a 90° bend. Furthermore, in compression mode, the sensor successfully resolved subtle pressure variations from handwriting (letters A‐F), generating distinct signal patterns that accurately reflected stroke number and structure (Figure 6j), demonstrating high sensitivity and the ability to decode complex motions.

Thus, the “gradient polarity modification” strategy serves as a versatile platform for imparting tailored functionalities to inert, pre‐formed SR devices, enabling their adoption in diverse advanced applications, including medical devices and soft electronics.

## 3 Conclusion

In summary, we have developed a versatile “gradient polarity modification” strategy that successfully overcomes a fundamental challenge in materials science: the inherent incompatibility between hydrophobic SR and strongly polar hydrophilic materials. By establishing a stepwise polarity gradient, this strategy enables the in situ covalent integration of strongly polar ionic structures throughout the pre‐cured SR network. The resulting hydrophilic interdigitated network achieves a combination of key properties, including exceptional hydrophilicity, aqueous lubricity, strain‐sensing capability, and functional designability. Furthermore, the incorporation of a Eu^3+^ complex as an intrinsic fluorescent probe provides a powerful tool to visually monitor the spatial progression of modification, offering unprecedented insight into the process. Critically, the strategy illustrates the potential for designable functionality, as evidenced by the successful modification with selected alkylating agent pairs. The successful modification of complex devices, including lubricative/antibacterial catheter and long‐lasting lubrication meniscus, directly underscores the method's technical viability and its superior capacity for creating multifunctional materials. By addressing the core issue of polarity mismatch, our strategy provides a pathway for obtaining advanced silicone‐based materials suited for applications in biomedical implants, soft robotics, and flexible electronics.

## Conflicts of Interest

The authors declare no conflicts of interest.

## Supporting information




**Supporting File**: advs75378‐sup‐0001‐SuppMat.docx.

**Supporting video 1**: advs75378‐sup‐0002‐Video S1.mp4.

**Supporting video 2**: advs75378‐sup‐0003‐Video S2.mp4.

## Data Availability

The data that support the findings of this study are available in the supplementary material of this article.
