# Supplementary material for: Integrated Hydrophilic Interdigitated Network for Silicone Rubber via a Gradient Polarity Modification Strategy
Source: Adv Sci (Weinh). 2026 Apr 20;13(40):e75378. doi: 10.1002/advs.75378 (PMC13335529; doi:10.1002/advs.75378)
Supplement: Supplementary file 1 — Supporting File: advs75378‐sup‐0001‐SuppMat.docx. [file ADVS-13-e75378-s003.docx]

Supporting Information

Integrated Hydrophilic Interdigitated Network for Silicone Rubber via a Gradient Polarity Modification Strategy

Lihui Zhang^A,B^, Rui Wang^B,C^, Pingru Su^A^, Yao Kou^A^, Zhengfeng Ma*^B,C^, Hui Tang^C^, Mingying Wang^C^, Lixin Wang^C^, Chunyu Zhou^C^, Shuanhong Ma^B^, Yu Tang*^A^, Feng Zhou^B^

1. Lihui Zhang, Pingru Su, Yao Kou, Yu Tang

State Key Laboratory of Applied Organic Chemistry, Key Laboratory of Nonferrous Metal Chemistry and Resources Utilization of Gansu Province, College of Chemistry and Chemical Engineering, Lanzhou University, Lanzhou 730000, P.R. China

E-mail: [tangyu@lzu.edu.cn](mailto:tangyu@lzu.edu.cn;)

1. Lihui Zhang, Rui Wang, Zhengfeng Ma, Shuanhong Ma, Feng Zhou

State Key Laboratory of Solid Lubrication, Lanzhou Institute of Chemical Physics, Chinese Academy of Sciences, Lanzhou 730000, P.R. China

E-mail: [mazhf@licp.cas.cn](mailto:mazhf@licp.cas.cn)

1. Rui Wang, Zhengfeng Ma, Hui Tang, Mingying Wang, Lixin Wang, Chunyu Zhou,

Shandong Laboratory of Advanced Materials and Green Manufacturing at Yantai, Yantai Zhongke Research Institute of Advanced Materials and Green Chemical Engineering, Yantai 264006, P.R. China

**Tables of Contents**

[1. Material S3](#_Toc32271)

[2. Preparation of Europium Complex S3](#_Toc2514)

[3. Preparation of SR-TAF S4](#_Toc6593)

[4. Preparation of SR-TAF-AA S4](#_Toc27069)

[5. Modification of silicone rubber catheter (SRC-PDMAEMA-n-BuBr) S4](#_Toc28190)

[6. Modification of silicone meniscus (SR-MEN-PDMAEMA-PS) S5](#_Toc26861)

[7. Detection and characterization S5](#_Toc29146)

[8. Supplementary Figures S10](#_Toc30585)

[9. References S29](#_Toc8215)

## Material

The silicone rubber (SR) used for method validation was a commercially available product prepared from methylvinyl silicone rubber and fumed silica (approximately 33 wt%), with a tensile strength > 5 MPa and an elongation at break > 400%. For application validation, a medical silicone catheter (polydimethylsiloxane-based) and a silicone meniscus prepared from Sylgard 184 (a vinyl-terminated polydimethylsiloxane prepolymer from Dow Corning, that crosslinks via platinum-catalyzed hydrosilylation) were used. Europium(III) chloride hexahydrate (99.99% metals basis), benzophenone (99%) and 2-isopropylthioxanthone (98%) were obtained from Macklin Biochemical Technology Co., Shanghai, China. 2-thenoyltrifluoroacetone (≥98%) was obtained from Aladdin Biochemical Technology Co., Ltd., Shanghai, China. ‌2-(Dimethylamino)ethyl acrylate (DMAEA, ≥99%) and 2-(Diethylamino)ethyl methacrylate (DEAEMA, ≥98%) were obtained from Aladdin Biochemical Technology Co., Ltd., Shanghai, China. ‌2-(Dimethylamino)ethyl methacrylate‌ (DMAEMA, 99%, GC) was purchased from Guangzhou Zhuosheng Biotechnology Co., LTD. n-butyl bromide (n-BuBr), methyl 4-bromobutyrate (Br-MB, ≥98%, GC) and 1,3-propanesulfonate (PS, 99%) were from Macklin Biochemical Technology Co., Shanghai, China. Sodium hydroxide, ethyl acetate, ethyl alcohol, tetrahydrofuran and dimethyl sulfoxidewere were obtained from Lianlong (Tianjin) Pharmaceutical Co., Ltd. LB nutrient agar was purchased from Solarbio Life Sciences Co., Ltd. Escherichia coli (ATCC 25922) was obtained from Shanghai Luwei Technology Co., Ltd., and *staphylococcus aureus* (ATCC 25923) was from Shanghai fuxiang Biotechnology Co., Lte. Physiological saline (0.9%), purchased from Wuhan Pricella Biotechnology Co., Ltd., was dissolved in ultrapure water prior to use. Cell culture media, phosphate-buffered saline (PBS) and the live/dead cell viability/cytotoxicity assay kit (including Calcein AM and EthD-1) were obtained from Wuhan Servicebio Technology Co., Ltd.

## Preparation of Europium Complex

Europium(III) chloride hexahydrate (0.1 mol) was dissolved in ethanol (40 mL). Under magnetic stirring, a solution of 2-thenoyltrifluoroacetone (0.41 mol) in ethanol was slowly added, and the mixture was homogenized. The pH of the reaction mixture was then adjusted to 6-7 using NaOH-ethanol (1mol/L) solution added dropwise, followed by stirring at room temperature for 1 h. The mixture was subsequently heated to 60°C and stirred isothermally for 14 h. After the reaction was complete and the mixture had been allowed to cool to room temperature, the mixture was filtered, and the collected filtrate was concentrated under reduced pressure, yielding the target product.

## Preparation of SR-TAF

A photoinitiator system comprising benzophenone (99%, 0.50 g) and 2-isopropylthioxanthone (0.50 g) was fully dissolved in tertiary amine-functionalized acrylates (TAF, 100 g). Pre-cleaned and dried SR samples (diameter: 25 mm; thickness: 5 mm) were immersed in this solution and kept at room temperature under dark conditions for 14 h to allow thorough permeation of monomers into the silicone matrix. The samples were then subjected to UV irradiation (λ = 365 nm, P = 9 W × 4) to initiate free-radical grafting and polymerization of DMAEMA. The irradiation protocol consisted of 2 h frontal exposure, followed by 4 h rear exposure, and a final 2 h frontal exposure. After irradiation, the samples were immersed in ethyl acetate to dissolve and remove unbound surface polymer and residual monomers. The samples were subsequently dried in an oven at 60°C until constant weight was achieved, yielding TAF modified SR.

## Preparation of SR-TAF-AA

To prepare hydrophilically modified SR, the TAF modified SR was immersed in a tetrahydrofuran (THF) solution containing alkylating agent (AA, 25 g). To achieve visualization, 0.5 wt% of a fluorescent europium(III) complex was incorporated as a luminescent probe. The depth of penetration and the degree of functionalization were modulated by controlling the reaction time (S-1 for 30min, S-2 for 1h, S-3 for 2h, S-4 for 3h and S-5 for 5h). Upon completion, the samples were washed with ethanol to remove residual reactants. Finally, dried in an oven at 60°C for 48 h to eliminate solvent residues, affording synchronously surface-bulk hydrophilically modified SR. Following the same procedure described above, the alkylation reactions with n‑BuBr, Br‑MB, and PS in the applicability study were conducted at 60 ^o^C for 2 h.

## Modification of silicone rubber catheter (SRC-PDMAEMA-n-BuBr)

The silicone rubber catheter (SRC) was immersed in a DMAEMA solution containing a photoinitiator and stored at room temperature in the dark for 8 hours. The polymerization was subsequently initiated under UV light with an irradiation duration of 4 hours. The sample was then rinsed with ethyl acetate to remove unbound polymers and residual monomers, followed by drying in an oven at 60 °C. It was further treated in a THF solution containing n-BuBr (40 wt%) and [Eu(TTA)_4_]^–^ (0.5 wt%) at 60 °C for 2 hours to complete the quaternization reaction. Finally, the product was thoroughly washed with ethanol, dried at 60 °C, and denoted as SRC-PDMAEMA-n-BuBr.

## Modification of silicone meniscus (SR-MEN-PDMAEMA-PS)

A silicone rubber meniscus (SR-MEN) was prepared using Sylgard 184 with a base-to-curing agent ratio of 10:1, cured at 60 °C for 14 hours. The resulting meniscus was immersed in a DMAEMA solution containing a photoinitiator and stored in the dark at room temperature for 10 hours. Polymerization was initiated under UV irradiation for 4 hours. The sample was cleaned with ethyl acetate to remove unreacted polymer and residual monomers and dried at 60 °C. It was then reacted in a THF solution containing PS (40 wt%) at room temperature for 4 hours to accomplish the sulfonation reaction. After thorough washing with ethanol and drying at 60 °C, the final product was designated as SR-MEN-PDMAEMA-PS.

## Detection and characterization

**The test of MS**

Mass spectrometric analysis was carried out using a ACQUITY UPLC I-Class Plus-XEVO G3 QTOF mass analyzer. The concentration of the sample is 0.1 mg/mL. The emission slit width was set to 1 nm, and fluorescence was measured at an emission wavelength of 400 nm, while near-infrared emission profiles were recorded using a dedicated NIR-sensitive detection module.

**The test of NMR**

1H NMR spectra data were recorded at 298 K on Bruker JNM-ECS 400M spectrometer operating at 400 MHz nuclear magnetic resonance spectrometer using Methanol-d_4_ as the solvents. Chemical shifts are given in ppm with reference to the residual solvent peak of the deuterated solvents. Coupling constants (J) are presented in Hz.

**The test of UV spectra**

UV-Vis spectra were acquired on a UV-2600 UV-vis spectrophotometer (Shimadzu Corporation, Japan) . The absorption was measured across the 200-800 nm range against a solvent reference.

**The test of fluorescence properties**

The fluorescence properties were characterized using a HORIBA FL-3 spectrofluorometer with a flashing xenon lamp as the excitation source, using an excitation wavelength of 365 nm.

**The test of FTIR**

The chemical compositions of the europium complex (Eu^3+^) and SR samples before and after modification were characterized using Attenuated Total Reflectance Fourier Transform Infrared Spectroscopy (ATR-FTIR, Nicolet iS10, Thermo Scientific, USA). Spectra were recorded over the wavenumber range of 800-4000 cm^-1^ with a resolution of 4 cm^-1^.

**The test of SEM**

Morphological features of the materials were obtained using an Environmental Scanning Electron Microscope (ESEM, Quanta 650 FEG, FEI Company, Czech Republic). Sample preparation involved immersion in water for 7 days to achieve full water saturation and swelling, followed by freezing at -18 ^o^C for 24 hours and subsequent freeze-drying for 48 hours. Prior to imaging, sample surfaces were sputter-coated with a thin gold layer.

**The test of composition and water absorbing capacity**

Weight analysis was employed to quantitatively characterize the mass changes associated with each modification step. Pure SR (blank) was rapidly rinsed with ethanol and dried in an oven at 60 °C for 4 h. The dried sample was weighed using an analytical balance (ME 104E/02, Mettler Toledo), and the mass was recorded as m_SR_. For the preparation of tertiary amine-functionalized SR (SR-TAF), after the grafting reaction, the sample was immersed in ethyl acetate overnight to remove unreacted monomers and physically adhered polymers. It was then thoroughly washed with fresh ethyl acetate and dried in an oven at 80 °C to constant weight (with a difference between two consecutive weighings not exceeding 0.5 mg). The resulting mass was recorded as m_SR-TAF_, and the mass of grafted TAF was calculated based on the mass increase. The dried SR-TAF was subsequently subjected to alkylation. Immediately after the reaction, the sample was rinsed with ethanol and dried in an oven at 80 °C to constant weight, yielding the mass m_SR-TAF-AA_.

Water absorption was determined gravimetrically. Dried samples were fully immersed in DI water at room temperature, and their mass changes were monitored at designated time points over a 7‑day period. At each designated time point, the sample was taken out, lightly blotted with filter paper to remove surface-adhered water, and weighed to obtain mSR-_TAF-AA-W_. The amounts of grafted TAF (m_TAF，_Equation 1), alkyl chains (m_AA，_Equation 2), and absorbed water (m_W，_Equation 3) were calculated using the following equations:

$\text{m}_{TAF}=\text{m}_{SR-TAF}-\text{m}_{SR}$ (Equation 1)

$\text{m}_{\text{AA}}=\text{m}_{SR-TAF-AA}=\text{m}_{SR-TAF}$ (Equation 2)

$\text{m}_{W}=\text{m}_{SR-TAF-AA-W}-\text{m}_{SR-TAF-AA}$ (Equation 3)

**The test of surface contact angle**

Surface contact angles were measured at 23 ^o^C using an optical contact angle goniometer (DSA-100, Krüss GmbH, Germany). Prior to measurement after water immersion, surface-adhered water was removed using filter paper.

**The test of thermal properties**

Thermal properties were characterized using a Simultaneous Thermal Analyzer (STA 449 F3, Netzsch, Germany). Measurements were conducted from room temperature to 800 ^o^C under N_2_ atmosphere at a heating rate of 10 ^o^C/min.

**Test of mechanical properties and strain-dependent electrical properties**

Mechanical properties were evaluated with a universal testing machine (EZ-Test EZ-LX, Shimadzu, Japan) equipped with a 500 N load cell. Tests included uniaxial tension, tensile cycling, and compression cycling. A programmable electrometer (Keithley 6514, Tektronix, USA) was integrated for in-situ electrical signal monitoring during mechanical loading. Sample Preparation: Specimens were immersed in distilled water for 7 days to achieve equilibrium swelling. Tensile samples were sectioned to dimensions of 50×5×2 mm^3^; compression specimens were cylindrical (Ø≈30 mm, height=6.5 mm). Surface moisture was blotted with filter paper prior to testing.

**The test of friction**

Frictional behavior was assessed using a ball-on-disk tribometer (CSM Tribometer, Anton Paar) for SR networks. Samples were affixed to glass substrates and tested against a 6 mm-radius counterball under reciprocating motion (5 mm stroke length). Standard conditions included a 5 N normal load, 1 Hz frequency, and distilled water lubrication. Parametric studies examined load/frequency effects on friction. Wear resistance was quantified via 10,000-cycle endurance tests. Post-test wear tracks were characterized by optical microscopy (Olympus BX51). Adhesion forces between counterfaces (316L stainless steel, PP, Si_3_N_4_ balls) and materials were measured using an adhesion tester (MFT-3000, Rtec Instruments) under 1 N contact pressure (59 s dwell time).

**The test of antibacterial properties**

The antibacterial properties of the material were evaluated using *Escherichia coli* and *Staphylococcus aureus* as indicator strains. Initially, the revived bacterial strains were incubated at 37 ^o^C for 12–16 hours. Subsequently, the resulting bacterial suspension was diluted with sterile physiological saline to a concentration of approximately 10^7^ CFU/mL. A 1 mL aliquot of this bacterial suspension was used to immerse the test samples (SR and SR-DMAEMA-nBuBr：0.5 cm × 0.5 cm；SRD and SRD-DMAEMA-nBuBr：Φ 0.75 cm × 1.0 cm), followed by contact incubation at 37 ^o^C for 5 hours. After incubation, the sample was removed and gently rinsed three times with sterile physiological saline to remove loosely adhered bacteria from the sample surface. The sample was then transferred into 2 mL of sterile physiological saline and subjected to ultrasonic treatment in an ultrasonic cleaner for 3 minutes to facilitate the detachment of firmly adhered bacteria into the solution. The saline solution containing the detached bacteria was diluted 10-fold, and an 100 μL of the diluted solution was pour-plated onto LB nutrient agar plates. The plates were incubated at 37 ^o^C in a constant-temperature incubator for 24 hours. The number of viable bacterial cells remaining on the sample surface was quantitatively assessed by enumerating the Colony Forming Units (CFU) formed on the plates.

**The test of XPS**

X-ray photoelectron spectroscopy (XPS) was performed using an ESCALAB 250Xi spectrometer (ThermoFisher Scientific, USA) to characterize the chemical composition and structural evolution during the modification process of SR.

**The test of cytotoxicity**

The cytotoxicity of the materials was evaluated using an extraction-based method. Specifically, 400 mg of Sylgard 184 silicone-both before and after hydrophilic modification-was sterilized via UV irradiation, immersed in 2 mL of serum-containing cell culture medium, and incubated at 4 ^o^C for 24 hours. The supernatant was collected after extraction for subsequent assays, with fresh complete medium used as the negative control. L-929 mouse fibroblast cells were diluted and seeded into 96-well plates at a density of approximately 5×10^4^ cells per well. The cells were cultured in fresh complete medium at 37 ^o^C for 24 hours. After aspiration of the medium, 100 μL of the material extract was added to each well. The negative control group received fresh complete medium. All experimental and control conditions were performed in triplicate. The plate was returned to the incubator for another 24 hours. A working solution for live/dead staining was prepared by diluting 0.5 μL of Calcein-AM and 2 μL of EthD-1 in 1 mL of PBS, followed by thorough mixing and protection from light. After careful removal of the culture medium, 100 μL of the staining solution was added to each well and incubated at 37 ^o^C in the dark for 10 minutes. The staining solution was then aspirated, and each well was washed with 100 μL of PBS before immediate imaging using an inverted fluorescence microscope (NIKON ECLIPSE Ti2-E inverted microscope, Nikon, Japan). Images were acquired from the green and red channels within the same field of view and subsequently overlaid using imaging software to generate composite images.

## Supplementary Figures


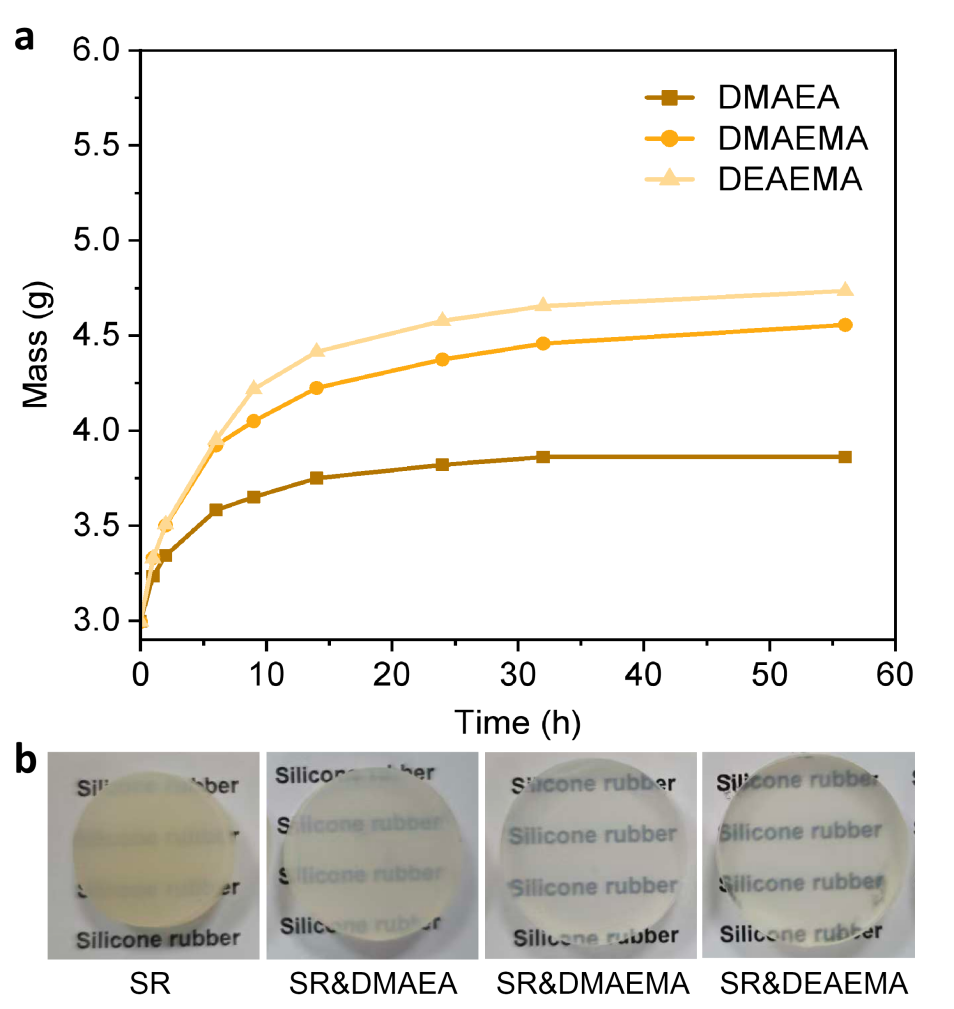


**Figure S1**. (a) Swelling curves of silicone rubber in different tertiary amine monomers; (b) Photographs of silicone rubber after swelling.

As shown in Figure S1 (a), the swelling curves of silicone rubber in different TAF monomers are presented. According to the like-dissolves-like principle, silicone rubber, as a nonpolar and highly hydrophobic polymer, exhibits permeability to monomers that primarily depends on the degree of polarity matching between the monomer and the matrix; specifically, monomers with lower polarity display higher permeability in silicone rubber. The permeability order of the three monomers follows DEAEMA > DMAEMA > DMAEA, which inversely correlates with their polarity order, indicating that the permeability behavior is dominated by differences in solubility. Among them, DEAEMA shows the highest permeability due to the presence of two ethyl groups on its amino moiety, which significantly enhances its hydrophobicity and affinity for silicone rubber. DMAEMA, bearing methyl groups on the amino moiety, exhibits weaker hydrophobicity than DEAEMA, resulting in moderate permeability. In contrast, DMAEA shows the lowest permeability. This can be attributed to its acrylate structure lacking an α-methyl group, which leads to greater exposure of the polar ester group, combined with the methyl-substituted amino group, rendering it the least hydrophobic and thus the least compatible with silicone rubber. Additionally, the three monomers have relatively small differences in molecular volume, which have a limited impact on the diffusion coefficient. This further supports that the observed differences in permeability are primarily governed by solubility variations arising from differences in polarity.

As depicted in the swelling curves, the swelling of silicone rubber in all three monomers levels off after 14 h of immersion, indicating that swelling approaches equilibrium. Concurrently, the transparency of the swollen silicone rubber increases significantly (Figure S1 (b)), which facilitates uniform UV light-triggered polymerization. Therefore, an immersion time of 14 h was selected as the pre-swelling duration to ensure adequate monomer permeation while providing optimal conditions for subsequent photopolymerization.


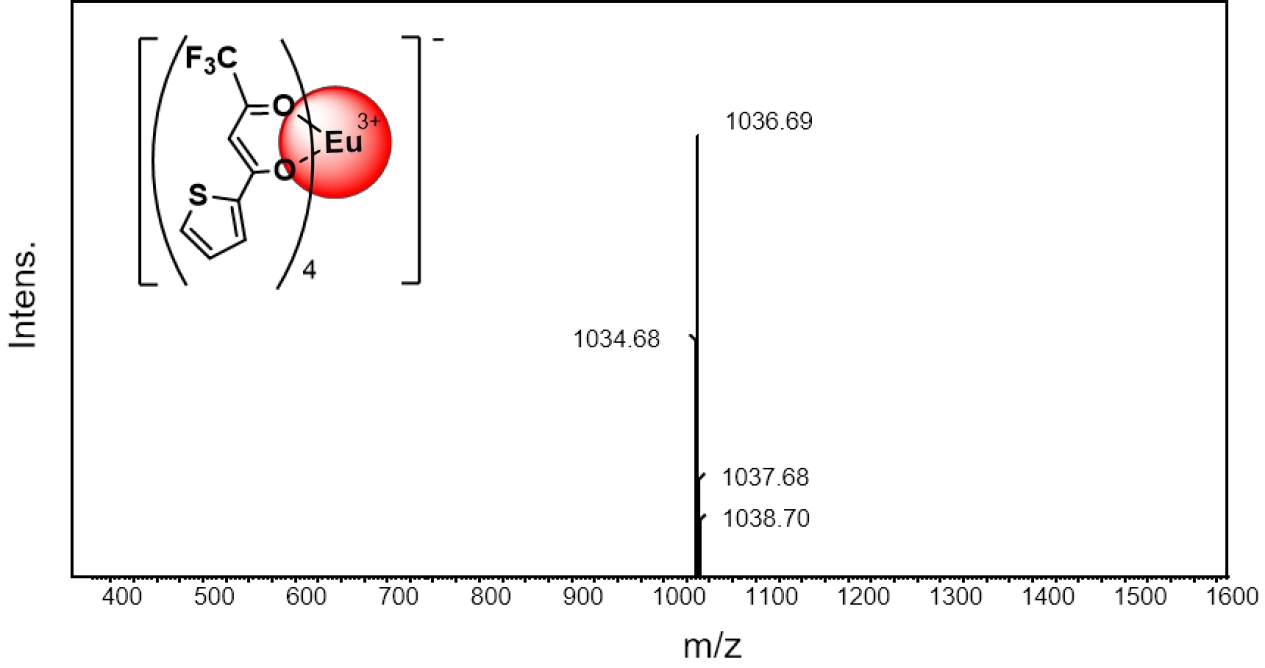


**Figure S2**. Mass spectrum of [Eu(TTA)_4_]^–^.

The molecular composition (Figure S2) of the [Eu(TTA)_4_]^–^ complex was unambiguously confirmed by mass spectrometry (MS), with the observed signal at m/z 1034.68 and 1036.69^[S1]^.

**
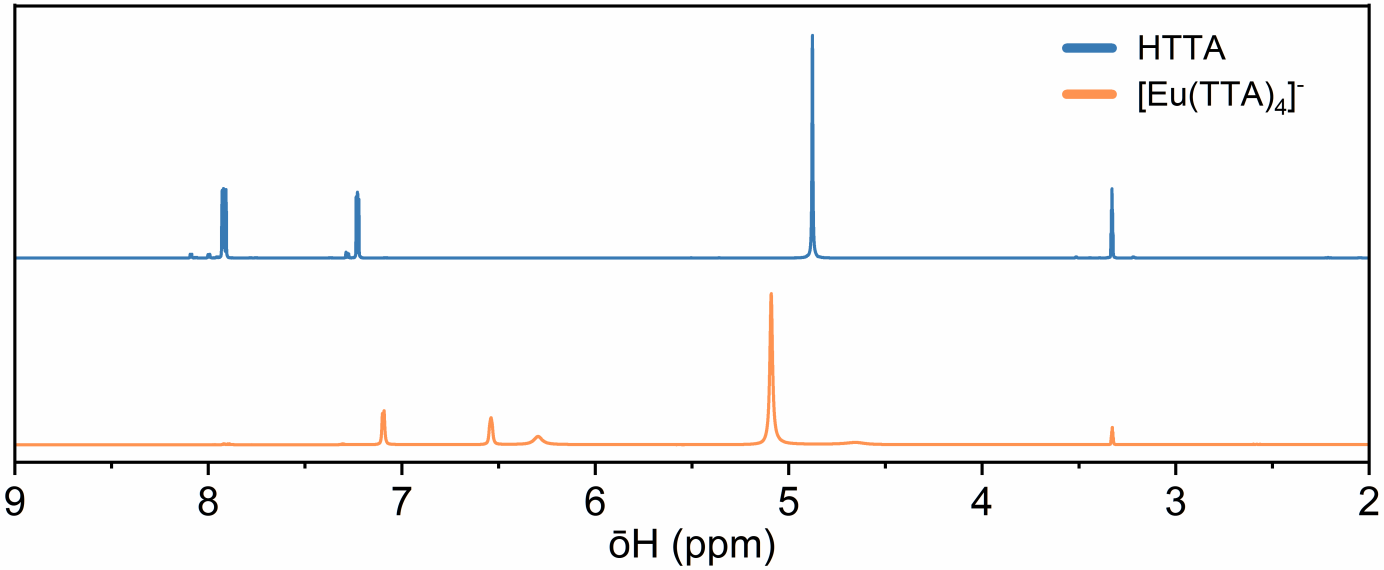
**

**Figure S3**. NMR spectrum of [Eu(TTA)_4_]^–^.

The ¹H NMR spectrum of the HTTA and [Eu(TTA)_4_]^–^ complex (Figure S3) confirms the successful coordination of the TTA. The aromatic protons of HTTA at 7.9 ppm and 7.2 ppm underwent a dramatic shift to 7.1 ppm and 6.5 ppm upon coordination. This significant change confirms the formation of the complex.


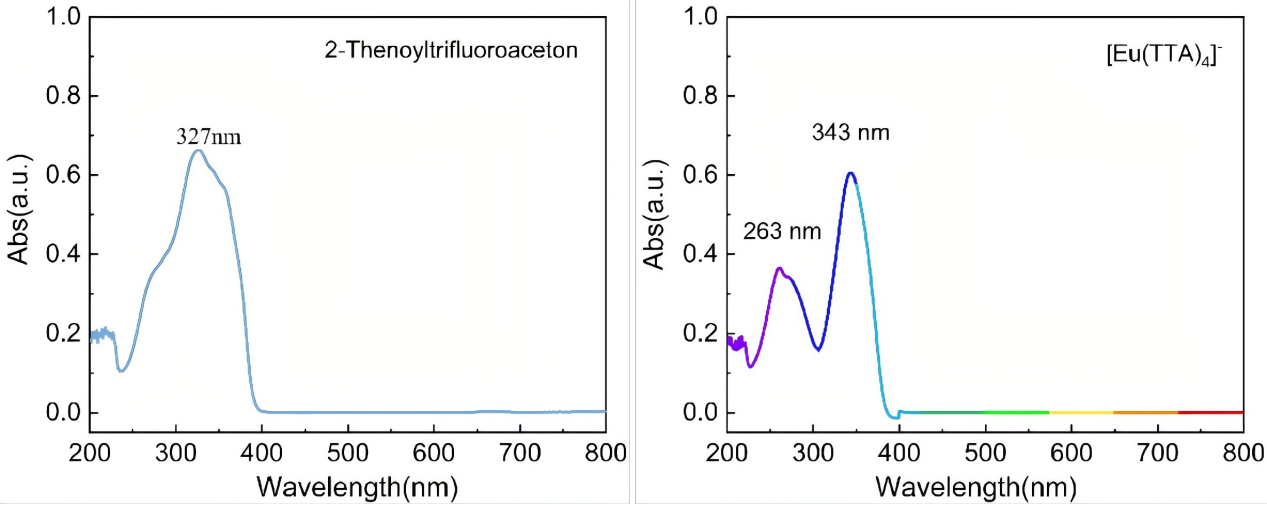


**Figure S4**. UV spectra: (a) ligand; (b) [Eu(TTA)_4_]^-^.

The ligand 2-thenoyltrifluoroacetone shows a broad absorption band in the range of 250–370 nm (Figure S4a). The formed complex [Eu(TTA)_4_]^–^ displays two distinct absorption peaks at 263 nm and 343 nm (Figure S4b). The UV-vis absorption spectrum provides definitive evidence for the successful synthesis of the [Eu(TTA)_4_]^–^ complex. The non-superimposable spectral features, including a bathochromically shifted band at 343 nm, are ascribed to ligand coordination and the emergence of a ligand-to-metal charge transfer (LMCT) state. This confirms the ligands function as an effective "antenna" for sensitizing Eu^3+^ emission ^[S2]^.


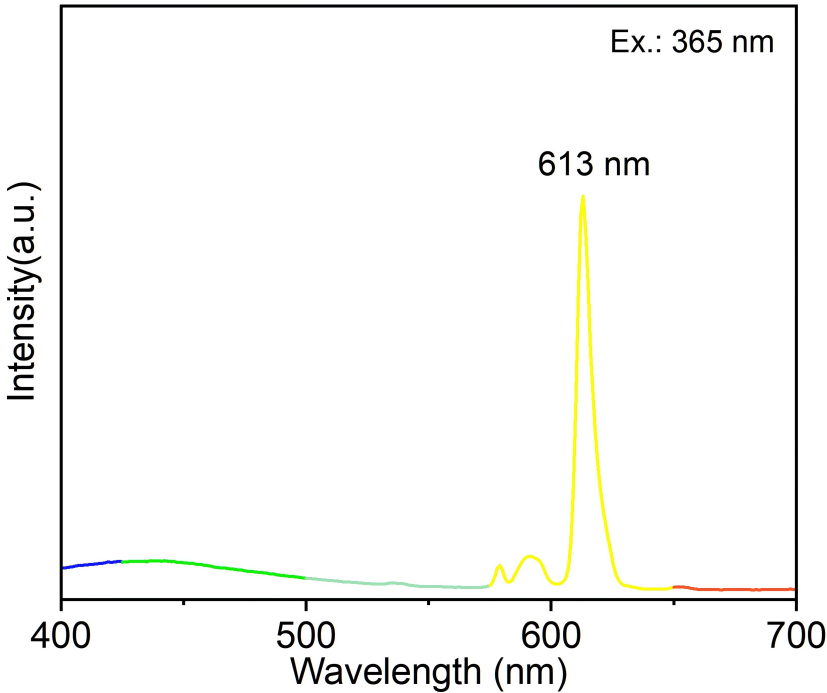


**Figure S5**. Fluorescence emission spectrum of [Eu(TTA)_4_]^–^.

Fluorescence emission spectrum (Figure S5) under 365 nm excitation showed a intense emission peak at 613 nm, corresponding to the characteristic red emission of Eu^3+^ (^5^D_0_→^7^F_2_ transition) ^[S3]^.


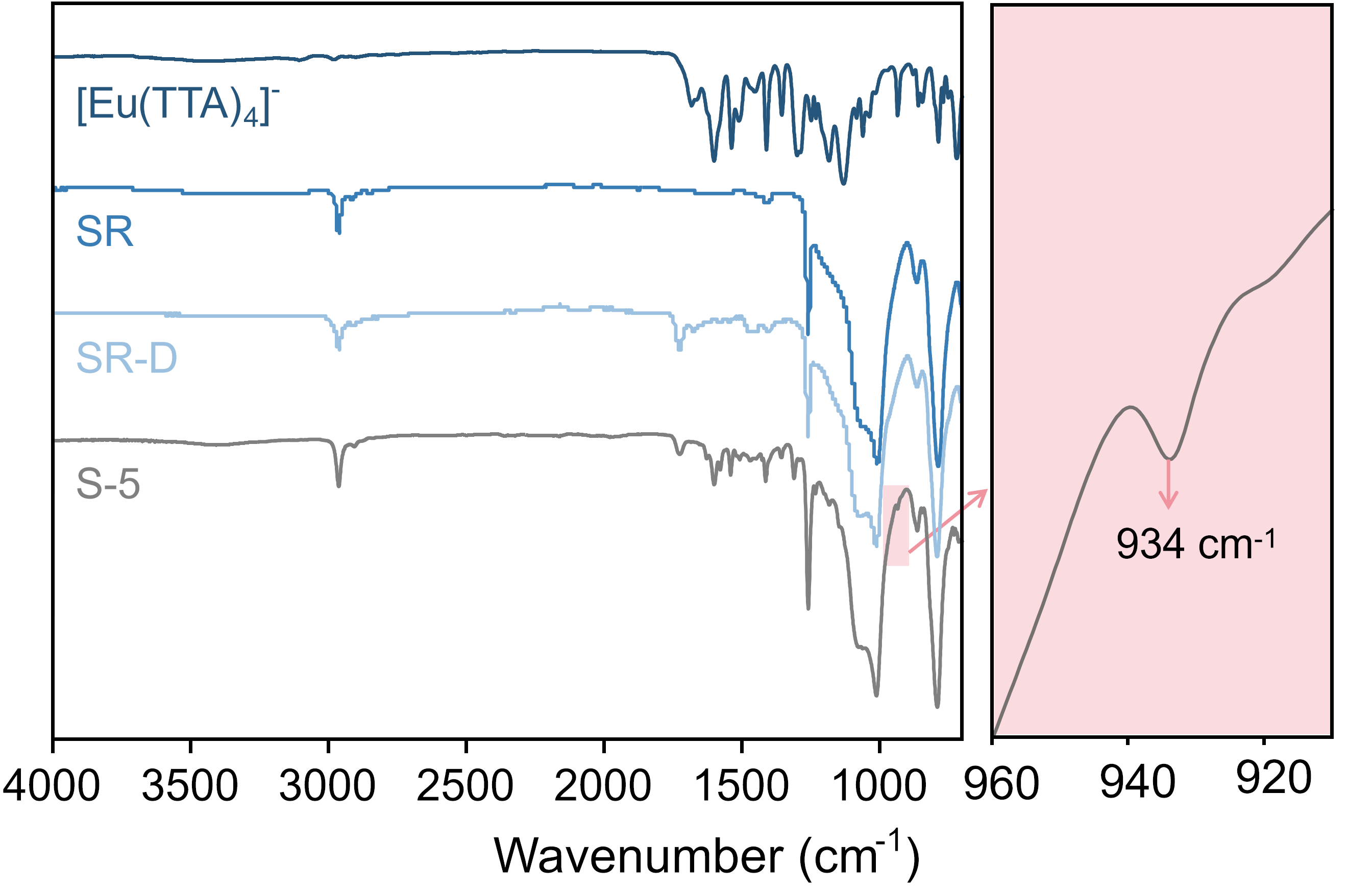


**Figure S6**. FTIR of [Eu(TTA)_4_]^–^ and the modified networks.

The FTIR spectrum of the [Eu(TTA)_4_]^–^ complex (Figure S6) shows the characteristic C=O stretching vibration at 1606 cm^-1^ and the C-F stretching band at 1350 cm^-1^, confirming the successful formation of the complex. FTIR spectra confirmed DMAEMA grafting by the appearance of a new C=O stretching vibration at 1750 cm^-1^. Characteristic peaks between 1350-1610 cm^-1^ in quaternized samples (e.g., S-5) indicated successful incorporation of the Eu(III) complex. Furthermore, a characteristic band assignable to the C-N^+^ stretching vibration of the quaternary ammonium group was observed at 934 cm^-1^, providing direct evidence for the formation of the quaternary ammonium structure.^[S4]^


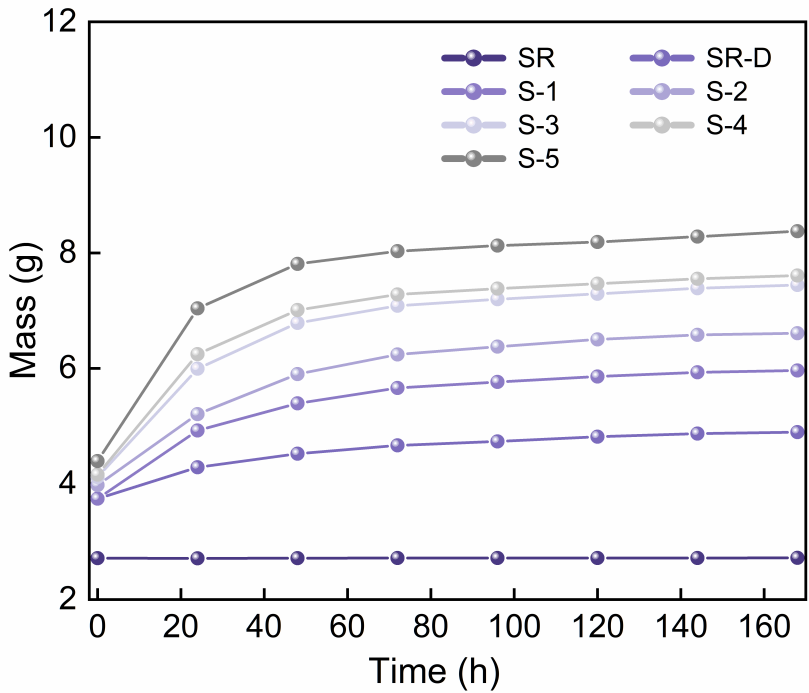


**Figure S7**. Time-dependent water uptake of SR and SR-DMAEMA-n-BuBr.

Water uptake (Figure S7), an indicator of material hydrophilicity, varies with chemical modification: hydrophobic silicone rubber (SR) exhibits negligible absorption, while polarized SR-D shows a modest uptake capacity. In contrast, alkylated derivatives demonstrate markedly enhanced water absorption, which correlates with the degree of alkylation. This trend is further accentuated with increasing alkylation, leading to a progressive rise in the water absorption rate. The water uptake largely reaches equilibrium within 72 hours.


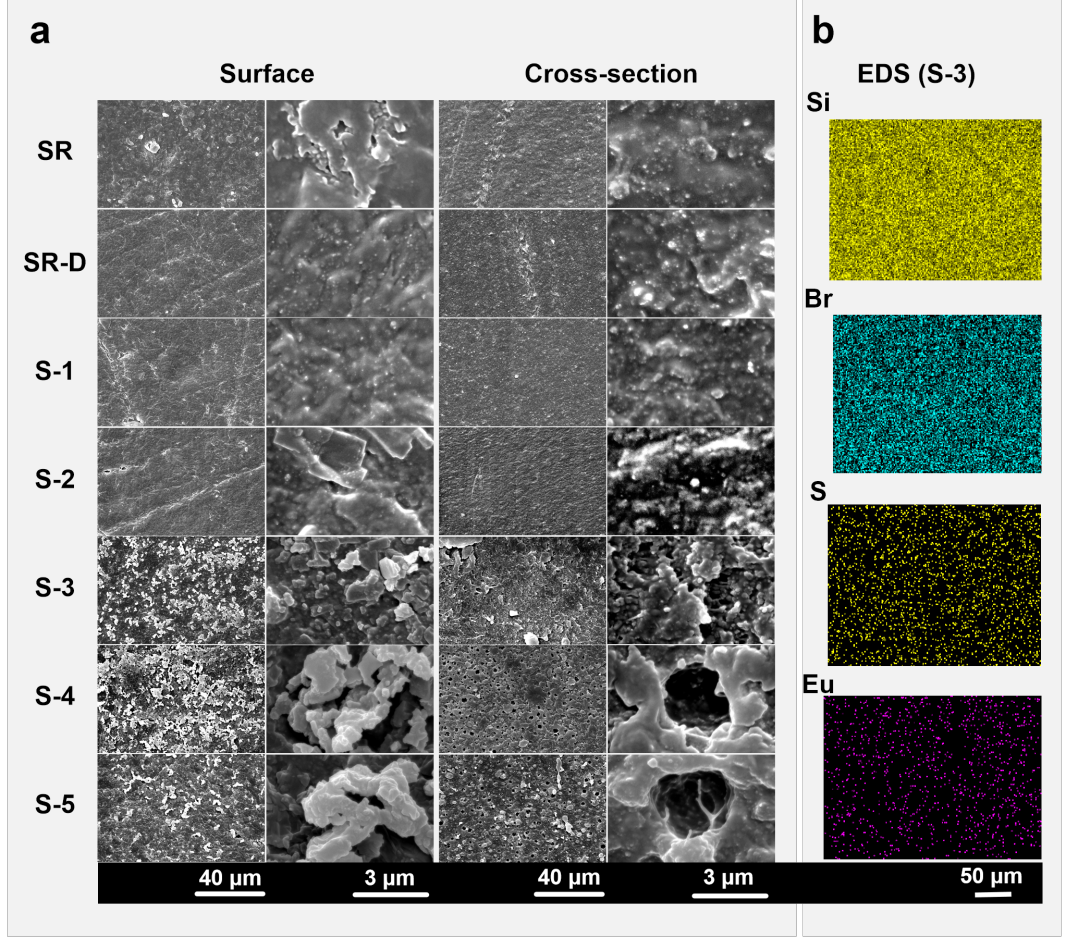


**Figure S8**. SEM (a) and EDS (b) images of SR and SR-DMAEMA-n-BuBr.

SEM (Figure S8a) and EDS (Figure S8b) analyses of the material surfaces and cross-sections directly reveal the material's microstructure. Alkylation progressively increased both the surface and internal roughness of the material, evolving into microporous structures after 2 h (sample S-3) and finally into channels 2-5 μm in diameter upon extended treatment (sample S-4, sample S-5), with the interiors filled with dendritic modified polymer. These structural changes are driven by the formation of a strongly hydrophilic quaternary ammonium structure following alkylation. The ensuing localized water accumulation facilitates channel development, thus enabling high water uptake and reflecting a substantial reorganization of the polymer network. EDS elemental mapping of sample S‑3 confirmed the homogeneous distribution of Si and Br, verifying uniform quaternization throughout the material. The co-localized signals of S and Eu further demonstrated the concurrent and uniform penetration of the fluorescent tracer with the quaternization agent.


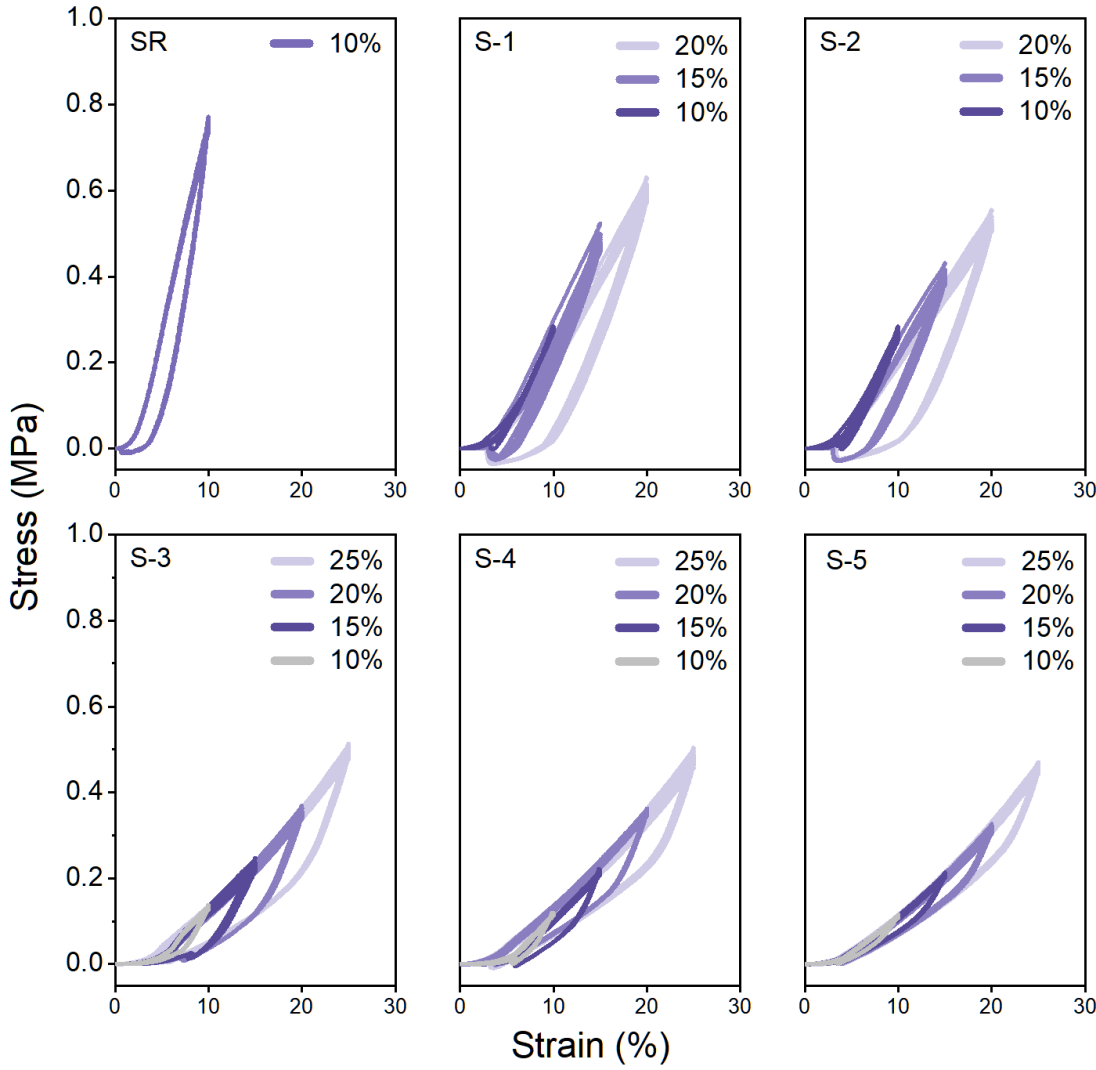


**Figure S9**. Cyclic compression performance test of SR and SR-DMAEMA-n-BuBr.

Compression cycling revealed deformation stability (Figure S9): while pristine silicone exhibited hysteresis at 10% strain, modified samples (S-2, S-3) showed nearly superimposed loading-unloading curves despite reduced modulus, indicating significantly lower energy dissipation attributed to water molecules lubricating chains and softening segments. However, at 15–20% compressive strain, enlarged yet reproducible hysteresis loops suggested enhanced internal friction during large deformations without substantial network damage.The reduction in the hysteresis loop area for the deeply alkylated sample indicates lower energy dissipation. This is due to its increased flexibility and high water content, which collectively reduce internal friction during deformation ^[S5]^.


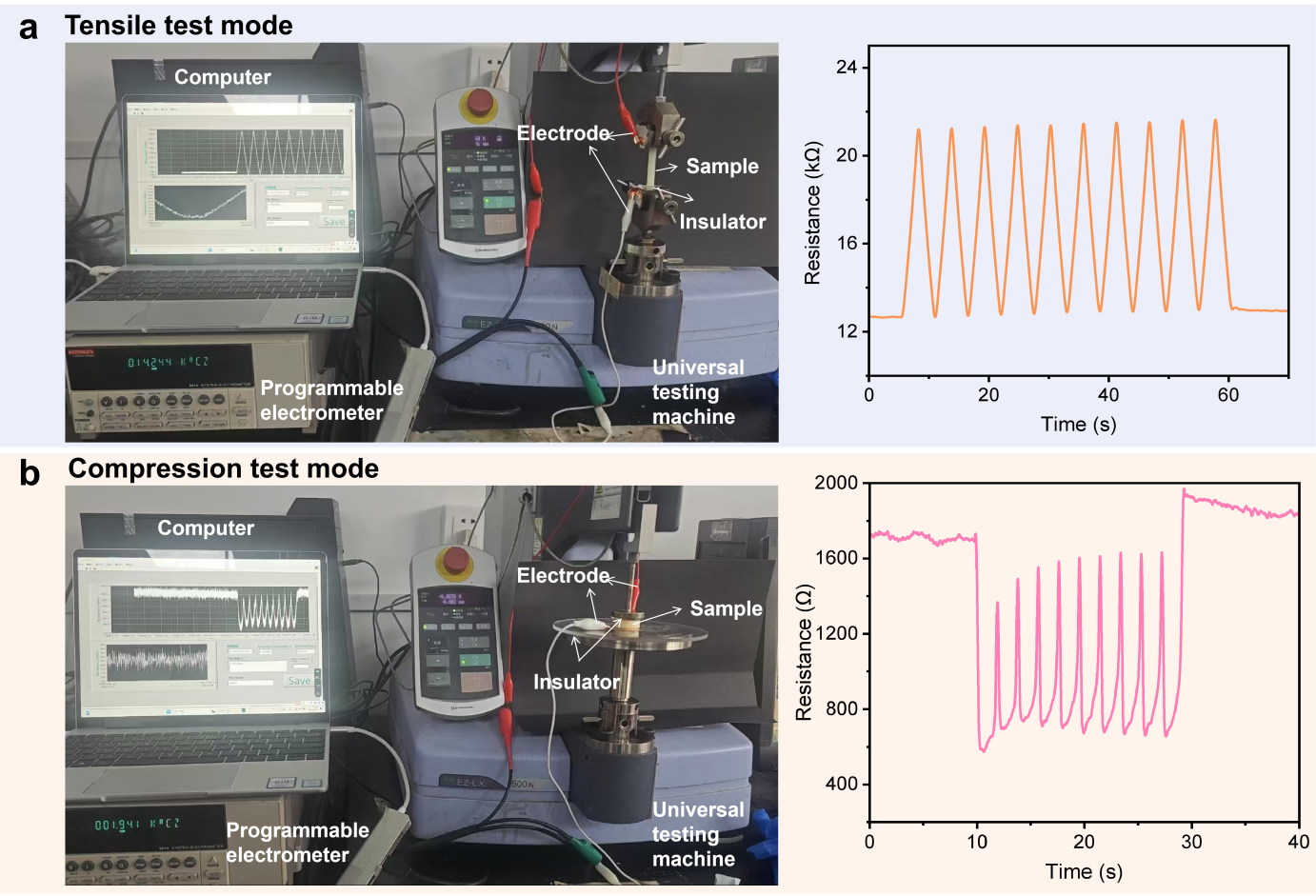


**Figure S10**. Strain electrical performance detection device and resistance variation under strain: tensile (a) and compressive(b) modes.

Figure S10 presents the strain electrical performance measurement setup and the resistance variation under strain in both tensile and compressive modes. The measurement system mainly consists of an electrometer, a universal tensile testing machine, and a computer. Figure S10(a) shows the resistance variation curve of the material under tensile mode. The initial resistance is 12.7 kΩ, which increases to 21.2 kΩ upon a tensile strain of 50%. Figure S10(b) shows the resistance variation curve under compressive mode. The initial resistance is 1710 Ω, which decreases to approximately 500 Ω at a compressive strain of 15%.


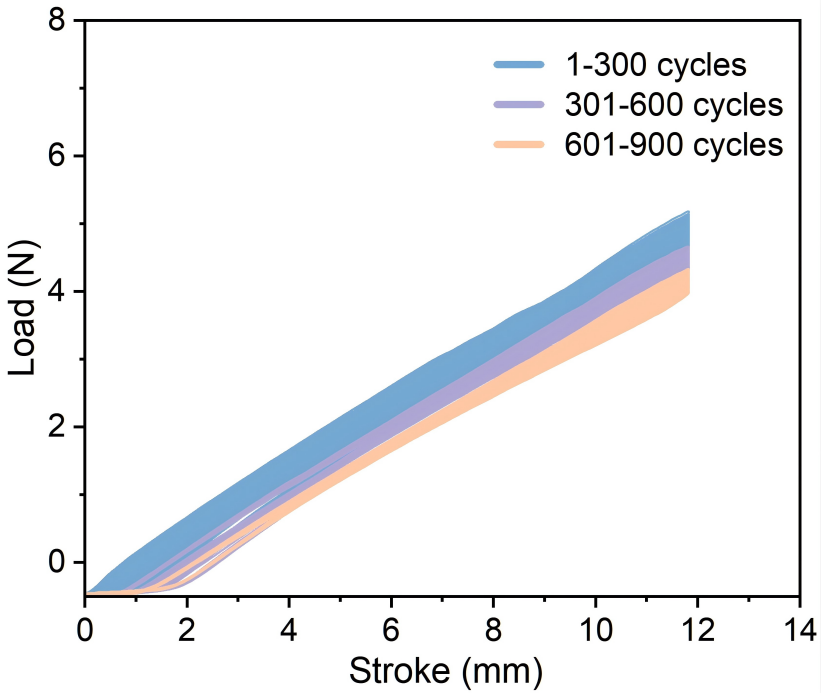


**Figure S11.** Cyclic test of tensile properties (1000 cycles).

The cyclic tensile behavior of sample S-3 (Figure S11) was evaluated over 1,000 cycles at 50% elongation. The material exhibited consistent elasticity with minimal degradation. The progressive shifting of the stress-strain curves, inferred from the data, may have been caused by slight grip slippage, a consequence of the reduced friction after hydrophilic modification.


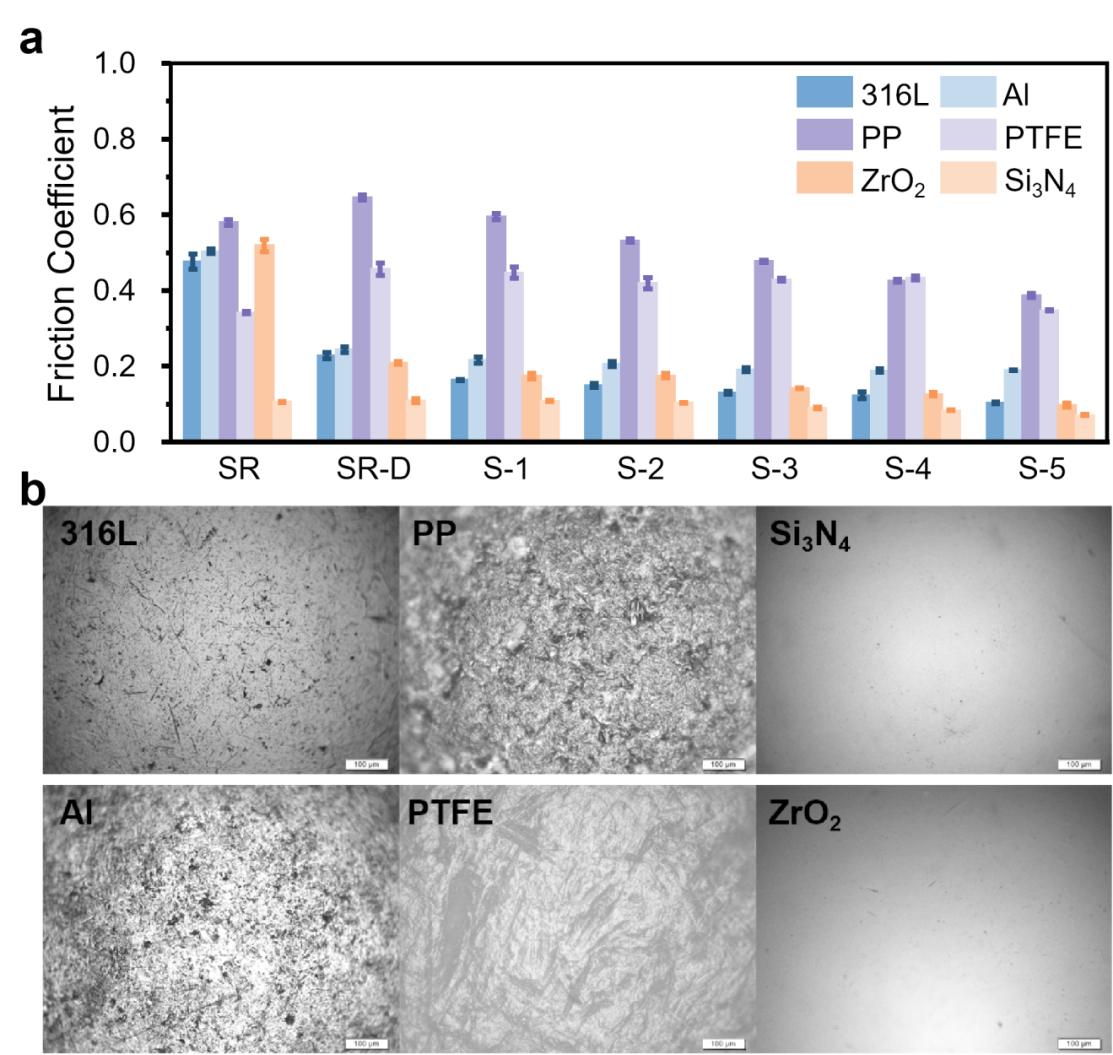


**Figure S12**. (a) COFs of hydrophilic interdigitated SR network with different counterfaces. (b) Surface micrograph of the friction pair ball.

The tribological behavior of pristine and modified SR was evaluated under water lubrication against six counterfaces (316L, Al, PP, PTFE, ZrO_2_, Si_3_N_4_). Pristine SR showed high friction against metals (COF: 0.48 for 316L, 0.50 for Al). DMAEMA grafting (SR-D) reduced COF to ~0.23-0.25. Quaternization further improved lubricity: initial sample S-1 gave COF of 0.16 (316L) and 0.22 (Al); prolonged quaternization (S-5) achieved optimal COF of 0.089 (316L) and 0.19 (Al). Against polymer counterfaces, pristine SR exhibited a high COF of 0.58 on PP due to strong adhesion and mutual hydrophobicity. PTFE showed lower initial COF (0.34) due to its self-lubricating property. DMAEMA modification induced hydration softening, raising COF to 0.64 (PP) and 0.46 (PTFE). Quaternization improved interfacial hydrophilicity, reducing COF by 15% on PP, but against hydrophobic PTFE, the reduction was limited (COF remained above pristine SR values) due to poor hydration layer formation. ZrO_2_ showed friction behavior similar to 316L. Si_3_N_4_ exhibited exceptional performance, with pristine SR COF of 0.10 (atomically smooth surface, moderate hydrophilicity, self-lubrication); optimal modification further reduced COF by 20% to 0.08.


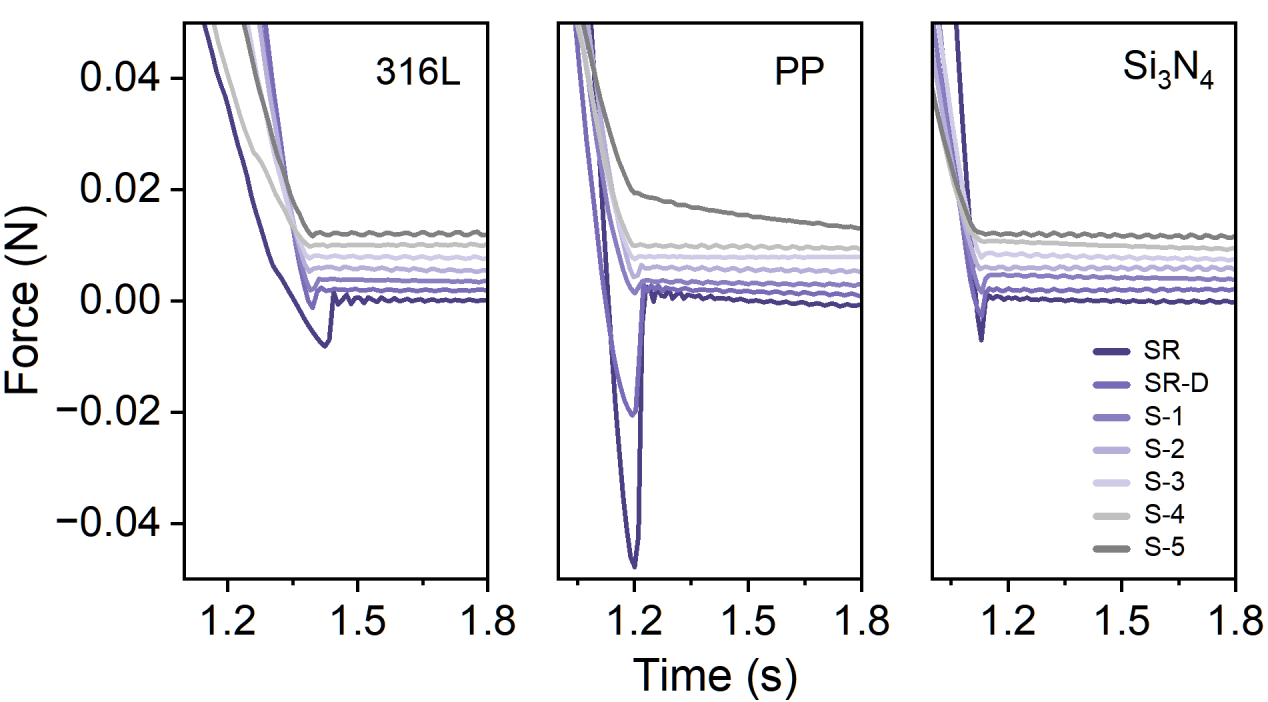


**Figure S13.** Adhesion between friction pair and SR before and after modification.

Adhesive behavior was observed between the silicone rubber and various counterfaces (Figure S13). The adhesive forces were relatively low for 316L alloy steel and Si₃N₄ ceramic, measuring 0.008 N and 0.007 N, respectively, while that for the PP pair was significantly higher at 0.048 N. Following hydrophilic modification, the adhesive forces for all three counterfaces were substantially reduced, due to the formation of an aqueous film on the modified surface, which functioned as a lubricating layer. Furthermore, this trend exhibited a correlation with the material's hydrophilicity, becoming increasingly pronounced as hydrophilicity increased.


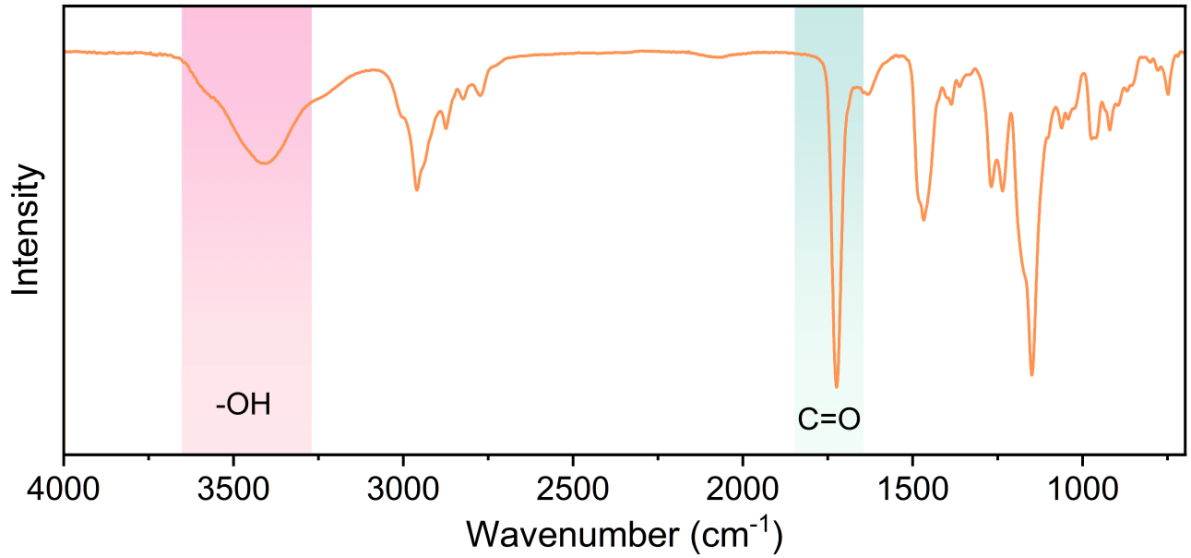


**Figure S14**. FTIR spectrum of exudate on the section (1mm).

FTIR spectrum (Figure S14) of collected exudate detected characteristic peaks at 1750 cm^-1^ (attributed to the C=O group in DMAEMA) and 3300 cm^-1^ (characteristic of water), confirming the hydrophilic polymer aqueous lubrication mechanism.


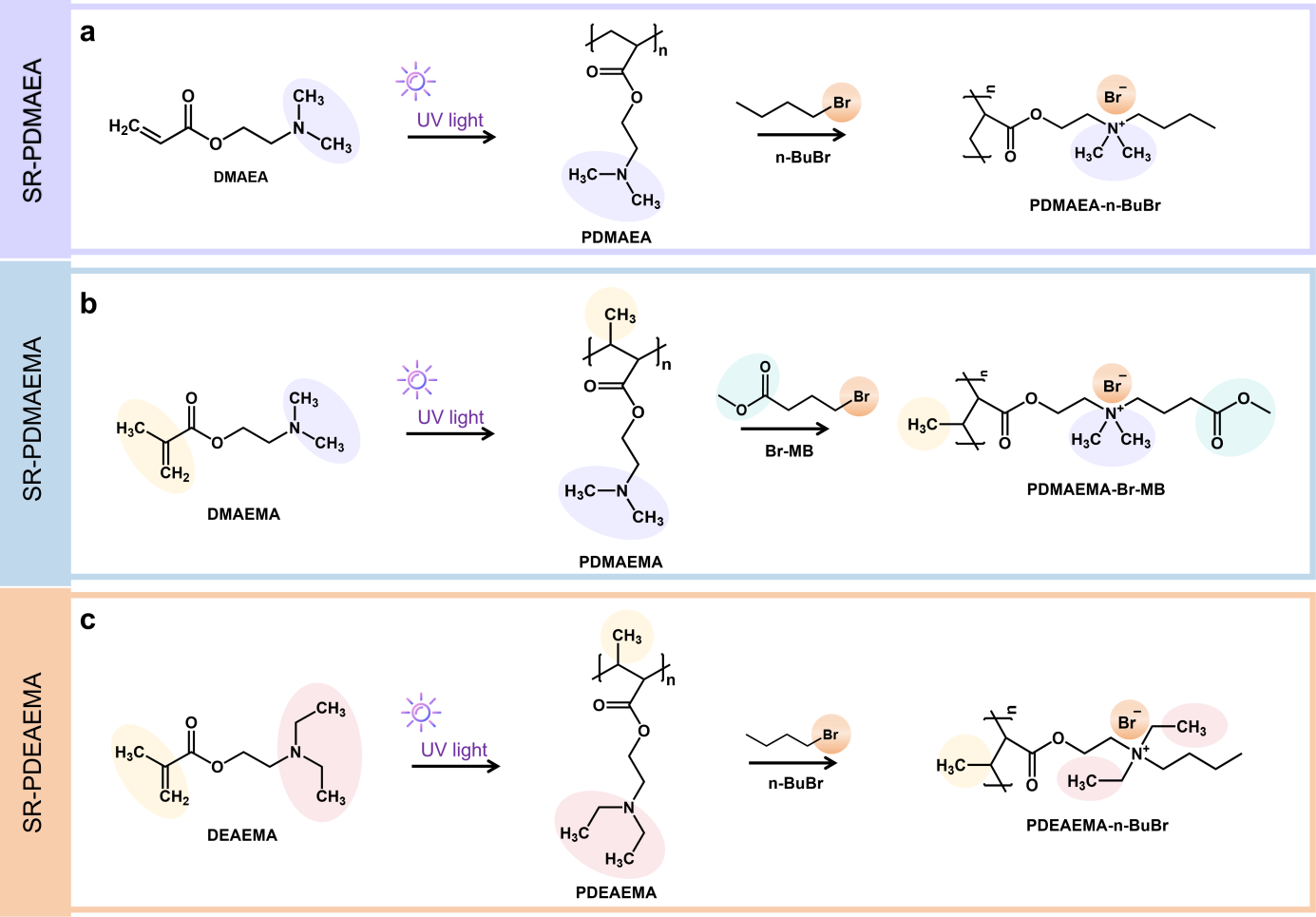


**Figure S15.** (a) Preparation of PDMAEA-n-BuBr modified SR. (b) Preparation of PDMAEMA-n-BuBr modified SR. (c) Preparation of PDEAEMA-n-BuBr modified SR.

Two additional TAF monomers, 2-(Dimethylamino) ethyl acrylate (DMAEA, Figure 15a) and 2-(Diethylamino) ethyl methacrylate (DEAEMA, Figure S15c) were selected for the tertiary amination modification of silicone rubber. The resulting materials were compared with SR-DMAEMA-n-BuBr.


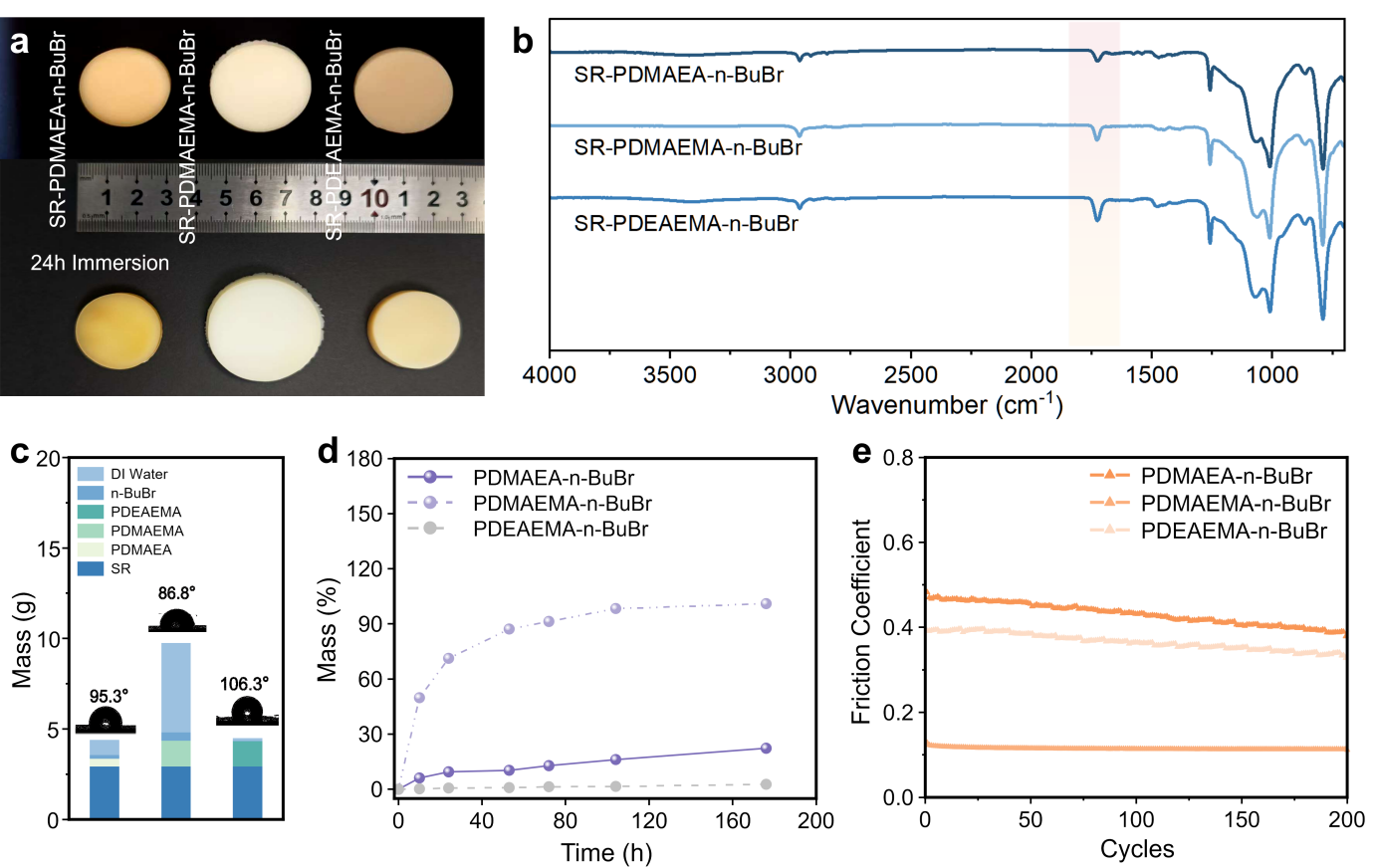


**Figure S16.** Performance test results of SR modified by different TAF monomers. (a) Photographs of SR modified with different TAF monomers before and after water absorption. (b) FTIR spectra of silicone rubber modified with different TAF monomers. (c) Composition and surface water contact angle of modified SR. (e) Water absorption performance of modified SR. (f) COFs of modified SR under water lubrication conditions.

SR was initially functionalized with three TAF monomers (DMAEA, DMAEMA, DEAEMA), followed by quaternization with n-BuBr. DMAEMA and DEAEMA exhibited higher grafting efficiency and greater volume increase. After quaternization, materials with higher hydrophilic content showed enhanced water absorption and swelling (Figure S16a-b). FTIR (Figure S16c) confirmed successful grafting of all three monomers (C=O peak at ~1750 cm⁻¹), but grafting efficiency varied considerably. DMAEA, with low permeability and poor radical stability of its acrylate double bond, gave a low grafting degree of 13.9 wt%. DEAEMA, showing infiltration and photopolymerization behavior comparable to DMAEMA, achieved a high grafting degree of 46.6 wt%. Polarity matching was critical for quaternization (Figure S16d-e). SR-PDMAEA had low tertiary amine content and insufficient polarity, resulting in poor n-BuBr infiltration and limited hydrophilicity (water absorption 22.3%, WCA 95.3°). For SR-PDEAEMA, despite the high grafting degree, the weak polarity and significant steric hindrance of the diethylamino group impeded n-BuBr infiltration and the quaternization reaction, yielding only marginal improvements in hydrophilicity (water absorption 2.5%, WCA 106.3°). Water lubrication (Figure S16f) performance directly correlated with hydrophilicity. Modifications with DMAEMA and DEAEMA reduced the coefficient of friction to only 0.43 and 0.36, respectively, showing limited improvement.


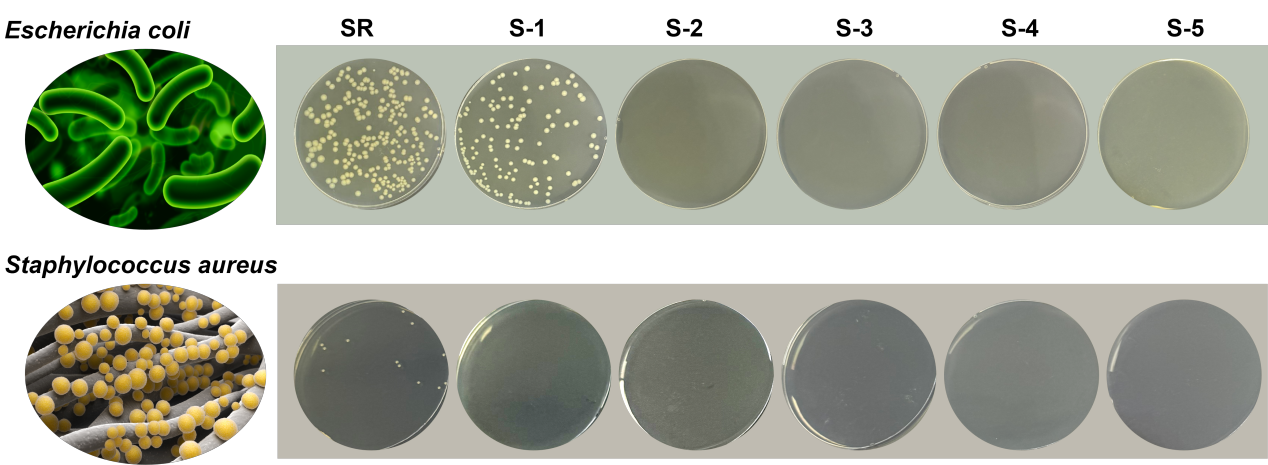


**Figure S17**. Antibacterial properties of SR and SR-DMAEMA-n-BuBr.

Superior antibacterial functionality is essential for materials in aqueous environments to prevent microbial corrosion and degradation. This study presents a hydrophilic silicone rubber modified with quaternary ammonium salts that confers enhanced antimicrobial activity (Figure S17). SR exhibited substantial bacterial colonization (194,400 CFU/cm^2^ for E. coli; 12,000 CFU/cm^2^ for S. aureus), the modified variants progressively inhibited growth. As shown in Figure S16, the modified materials exhibit potent antibacterial efficacy against *Escherichia coli* and *Staphylococcus aureus*. The pristine SR sustained substantial bacterial colonization, with counts reaching 194,400 CFU/cm^2^ for *E. coli* and 12,000 CFU/cm^2^ for *S. aureus*. Initial modification (S-1) significantly inhibited growth, reducing the counts to 112,000 CFU/cm^2^ for *E. coli* (a 42.4% reduction) and 0 CFU/cm^2^ for *S. aureus* (a 100% reduction). Progressive functionalization (S-2 to S-5) further enhanced surface hydrophilicity and antimicrobial ion density, culminating in the complete eradication (0 CFU/cm^2^) of both bacterial strains. This 100% bactericidal efficiency is attributed to a dual mechanism: enhanced bacterial contact due to improved hydrophilicity, and the electrostatic binding of quaternary ammonium cations to negatively charged microbial membranes, leading to disruption and cell death^[S6]^.

## References

[S1] T. B. Paolini, I. P. Assunçao, I. F. Costa, L. Blois, M. C. F. C. Felinto, R. T. Moura Jr., E. E. S. Teotonio, O. L. Malta, A. N. C. Neto, and H. F. Brito, “The influence of imidazolium counterions on the luminescence properties of Cnmim[Eu(tta)_4_] tetrakis complexes in solid-state and ionic liquid solutions,” *Journal of Luminescence* 263 (2023): 120158. https://doi.org/10.1016/j.jlumin. 2023. 120158

[S2] (a) Y. Kou, Y. Guo, L. Liang, X. Li, Y. Wang, P. Su, C.-H. Yan, and Y. Tang, “Electrostatic Self-Assembly of Ag-NPs Mediated by Eu^3+^ Complexes for Physically Unclonable Function Labels,” *Aggregate* 6, no. 3 (2025): e701. <https://doi.org/10.1002/agt2.701;> (b) P. Feng, X. Yang, X. Feng, G. Zhao, X. Li, J. Cao, Y. Tang, and C.-H. Yan, “Highly Stable Perovskite Quantum Dots Modified by Europium Complex for Dual-Responsive Optical Encoding,” *ACS Nano* 15, no. 4 (2021): 6266-6275. https://doi.org/10.1021/acsnano.0c09228

[S3] L. Liang, X. Yang, X. Yan, Y. Kou, Y. Zhang, P. Su, and Y. Tang, “Self-Assembly of Eu^3+^ Complexes Enabling Multistimuli-Responsive Data Encryption,” *Advanced Materials* 38, no. 2 (2025): e14252. https://doi.org/10.1002/adma.202514252

[S4] N. B. Colthup, L. H. Daly, and S. E. Wiberley, Introduction to Infrared and Raman Spectroscopy, 3rd ed., Boston: Academic Press, 1990. ISBN 978-0-12-182554-6. <https://doi.org/10.1016/C2009-0-21628-X>

[S5] (a) S. A. Chester and L. Anand, “A coupled theory of fluid permeation and large deformations for elastomeric materials,” *Journal of the Mechanics and Physics of Solids* 58, no. 11 (2010): 1879-1906. <https://doi.org/10.1016/j.jmps.2010.07.020;> (b) B. Barrière and L. Leibler, “Kinetics of solvent absorption and permeation through a highly swellable elastomeric network,” *Journal of Polymer Science Part B: Polymer Physics* 41, no. 2 (2003): 166-182. https://doi.org/10.1002/polb.10341

[S6] (a) L. Wang, Y. Pang, M. Xin, M. Li, L. Shi, and Y. Mao, “Effect of the structure of chitosan quaternary ammonium salts with different spacer groups on antibacterial and antibiofilm activities,”*International Journal of Biological Macromolecules* 276, Part 1 (2024): 133777. <https://doi.org/10.1016/j.ijbiomac.2024.133777>; (b) Z. Zhou, S. Zhou, X. Zhang, S. Zeng, Y. Xu, W. Nie, Y. Zhou, T. Xu, and P. Chen, “Quaternary Ammonium Salts: Insights into Synthesis and New Directions in Antibacterial Applications,” *Bioconjugate Chemistry* 34, no. 2 (2023): 302-325. https://doi.org/10.1021/acs.bioconjchem.2c00598
